# Inhibition of Aminotransferases by Aminoethoxyvinylglycine Triggers a Nitrogen Limitation Condition and Deregulation of Histidine Homeostasis That Impact Root and Shoot Development and Nitrate Uptake

**DOI:** 10.3389/fpls.2019.01387

**Published:** 2019-11-07

**Authors:** Erwan Le Deunff, Patrick Beauclair, Carole Deleu, Julien Lecourt

**Affiliations:** ^1^Normandie Université, UNICAEN, SF ICORE 4206, Caen, France; ^2^INRA Unité Expérimentale Fourrages Environnement Ruminants (FERLUS) et Système d’Observation et d’Expérimentation pour la Recherche en Environnement (SOERE), Les Verrines CS 80006, Lusignan, France; ^3^INRA—Agrocampus Ouest—Université de Rennes 1, UMR 1349 Institut de Génétique, Environnement et Protection des Plantes (IGEPP) Université de Rennes 1, Rennes, France; ^4^NIAB EMR, Crop Science and Production Systems New Road, East Malling, United Kingdom

**Keywords:** Aminoethoxyvinylglycine, tryptophan aminotransferase, histidine catabolism, aminocyclopropane-1-carboxylate synthase, Brassica napus, nitrate uptake, root morphogenesis, general amino acid control

## Abstract

**Background and Aims:** Although AVG (aminoethoxyvinylglycine) is intensely used to decipher signaling in ethylene/indol-3-acetic acid (IAA) interactions on root morphogenesis, AVG is not a specific inhibitor of aminocyclopropane-1-carboxylate synthase (*ACS*) and tryptophan aminotransferase (*TAA*) and tryptophan aminotransferase related (*TAR*) activities since it is able to inhibit several aminotransferases involved in N metabolism. Indeed, 1 mM glutamate (Glu) supply to the roots in plants treated with 10 μM AVG partially restores the root growth. Here, we highlight the changes induced by AVG and AVG + Glu treatments on the N metabolism impairment and root morphogenetic program.

**Methods:** Root nitrate uptake induced by AVG and AVG + Glu treatments was measured by a differential labeling with ^15^NO_3_^-^ and ^15^Nglutamate. In parallel a profiling of amino acids (AA) was performed to decipher the impairment of AA metabolism.

**Key Results:** 10 μM AVG treatment increases K^15^NO_3_ uptake and ^15^N translocation during root growth inhibition whereas 10 μM AVG + 1 mM ^15^Nglutamate treatment inhibits K^15^NO_3_ uptake and increases ^15^Nglutamate uptake during partial root growth restoration. This is explained by a nitrogen (N) limitation condition induced by AVG treatment and a N excess condition induced by AVG + Glu treatment. AA levels were mainly impaired by AVG treatment in roots, where levels of Ser, Thr, α-Ala, β-Ala, Val, Asn and His were significantly increased. His was the only amino acid for which no restoration was observed in roots and shoots after glutamate treatment suggesting important control of His homeostasis on aminotransferase network. Results were discussed in light of recent findings on the interconnection between His homeostasis and the general amino acid control system (GAAC) in eukaryotes.

**Conclusions:** These results demonstrate that AVG concentration above 5 μM is a powerful pharmacological tool for unraveling the involvement of GAAC system or new N sensory system in morphological and metabolic changes of the roots in leguminous and non-leguminous plants.

## Introduction

Inhibitors of aminotransferases such as aminoethoxyvinyl-glycine (AVG), Rhizobitoxine and aminooxyacetic acid (AOA) have recently been demonstrated to be non-specific, possessing a broad inhibitory spectrum (Berkowitz et al., 2006; Soeno et al., 2010; Le Deunff and Lecourt, 2016; Le Deunff, 2018). This offers the possibility to highlight the effects of nitrogen metabolism impairment on the root and shoot morphogenetic program and root nitrate uptake (Le Deunff and Lecourt, 2016; Le Deunff et al., 2016). Indeed, nitrogen nutrition and plant development depend not only on nitrate uptake and reduction, but also on the assimilation of nitrogen into amino acids, nucleic acid bases, proteins, polyamines, chlorophylls and hormones due to the action of many aminotransferases downstream the *GS/GOGAT* cycle (Stitt et al., 2002; Lea and Azevedo, 2006). Aminotransferases are the key enzymes involved in nitrogen (N), sulphur (S) and carbon (C) shuttling and distribution in plants through the interconversions between organic acids and amino acids (Christen and Mehta, 2001; Liepman and Olsen, 2004; Le Deunff, 2018). They catalyze amino group transfers from amino donor to amino acceptor. For their catalytic activity, they require pyridoxal-5’-phosphate (PLP) a phosphorylated form of vitamin B_6_ that is a versatile coenzyme. PLP biosynthesis depends on the glutamine produced by the *GS/GOGAT* cycle, glyceraldehyde 3-phosphaste (G3P) and ribose 5-phosphate (5RP) provided by the glycolytic and pentose phosphate pathways, respectively (Tambasco-Studart et al., 2007; Colinas et al., 2016). Aminotransferases have been classified in four families: alpha (α), beta (β), D-alanine aminotransferase and alanine racemase (Mehta et al., 1993; Christen and Mehta, 2001). The aminotransferases belonging to the α family have been further subdivided into four evolutionary subgroups (I–IV) based on their protein sequences and catalytic site structure (Mehta et al., 1993; Christen and Mehta, 2001). The subgroup I contains major enzymes involved in N, S and C assimilation and shuttling such as aspartate, alanine, tyrosine, histidine-phosphate and phenylalanine aminotransferases (Christen and Mehta, 2001). Interestingly, key enzymes involved in auxins (IAA and PAA) and ethylene biosynthesis such as tryptophan aminotransferase of *Arabidopsis* (*TAA1*), tryptophan aminotranferase related *(TARs*) and 1-aminocyclopropane-1-carboxylate synthase (*ACS*), respectively, belong also to the subgroup I of aminotransferases (Christen and Mehta, 2001; Stepanova et al., 2008; Tao et al., 2008; Sugawara et al., 2015). For example, X-ray structure analysis of *ACS* catalytic site showed that AA surrounding the active site share the same function as aspartate aminotransferase (*AAT*) counterpart (Tarun and Theologis, 1998; Capitani et al., 1999).

Despite the essential role played by *TAA*, *TARs* and *ACS* aminotransferases in the root morphogenetic program (Rahman et al., 2000; Stepanova et al., 2008; Tao et al., 2008; Okamoto et al., 2008; Yamada et al., 2009; Ma et al., 2014; Sugawara et al., 2015), the involvement of other aminotransferases within subgroup I of the α family on root development has been overlooked (Le Deunff et al., 2016; Le Deunff, 2018). *Arabidopsis* mutants of aminotransferases of N metabolism such as aspartate aminotransferase (*AAT*), alanine aminotransferase (*AlaAT*) and histidinol phosphate aminotransferase (*HPA*) displayed root developmental defects (Schultz et al., 1998; Miesak and Coruzzi, 2002; McAllister and Good, 2015; Mo et al., 2006). In *Arabidopsis* a comparison of the three *aat2-1, att2-2 and att2-3* mutants deficient in *AAT2* cytosolic isoform, found that only the *aat2-2* mutant showed a dramatic reduction in root length that was exaggerated after an addition of 20 mM of aspartate in the medium (Schultz et al., 1998; Miesak and Coruzzi, 2002). Alanine aminotransferase catalyzes the reversible conversion of alanine and 2-oxoglutarate into glutamate and pyruvate. In *Arabidopsis*, the *alaat1;2* double knockout mutants showed a significant reduction in the tap root length and primary lateral roots, whereas over-expression of *AlaAT* variants from diverse plant sources and different subcellular locations significantly increased root development at non-limiting and limiting external nitrate concentrations (McAllister and Good, 2015). The mutant *hpa1* carries a mutation in one of the two plastidial histidinol phosphate aminotransferases (*HPA*) that converts imidazoleacetol-phosphate and glutamate to histidinol-phosphate and α-ketoglutarate. The mutant presents a 30% reduction in root His content and displays defect in root meristems maintenance and reduction in the primary root and lateral roots development (Mo et al., 2006).

Furthermore, the use of non-specific inhibitors of aminotransferases such as AVG at a concentration greater than 5 μM has revealed that the impairment of N metabolism affects the root morphogenetic program and N uptake (Leblanc et al., 2008; Lemaire et al., 2013). Thus, in *Brassica napus*, the reduction of root elongation and root hairs formation by AVG treatment were counteracted by a significant increase of nitrate uptake and *BnNrt2.1* nitrate transporter gene expression (Leblanc et al., 2008). In comparison to controls, AVG-treated plantlets showed significant increases in the root levels of primary AA such as glutamate, glutamine, aspartate and asparagine (Beauclair et al., 2009; Lemaire et al., 2013). Moreover, root elongation was almost completely restored with 1 mM glutamate treatment in 10 μM AVG-treated plantlets suggesting that major aminotransferases of N metabolism were strongly impaired (Leblanc et al., 2008). Taken together, these results confirm that AVG is a non-specific suicide inhibitor of many PLP-dependent enzymes that belongs to the subgroup I of aminotransferases (Werck-Reichhart et al., 1988; Berkowitz et al., 2006; Soeno et al., 2010; Lin et al., 2010; Le Deunff et al., 2016). Owing to its broad inhibitory spectrum, there is a need to analyse responses of 10 μM AVG treatment on the root growth and development (Soeno et al., 2010). For example, it is well known that treatment with ACC (aminocyclopropane-1-carboxylate), the ethylene precursor, in AVG-treated plantlets does not restore the AVG-induced effect on root hair and primary root elongation. This suggests that other aminotransferases, in addition to *ACS* and *TAA*, are also targets for AVG and potentially involved in inhibition of the root morphogenetic program through N sensing systems such as TOR and GCN2 (General Control Non-derepressible-2) kinases (Leblanc et al., 2008; Soeno et al., 2010; Le Deunff et al., 2016; Le Deunff, 2018).

Otherwise, AVG and rhizobitoxine belong to the enol ether family and are a subclass of naturally occurring α-vinylic amino acids (Berkowitz et al., 2006; Boibessot et al., 2016) that are secreted by symbiotic rhizobacteria and pathogenic bacteria (Pruess et al., 1974; Owens et al., 1968; Mitchell et al., 1986; Yasuta et al., 2001). If these two natural compounds at low concentrations (< 5 μM) are known as inhibitors of two enzymes of methionine pathway involved in ethylene biosynthesis: cystathionine β-lyase and ACC synthase (Clausen et al., 1997; Yasuta et al., 1999; Rahman et al., 2000; Berkowitz et al., 2006), the role of their wide inhibitory spectrum on aminotransferases of N metabolism has been neglected in establishing symbiosis and increasing the nodule number (Peters and Crist-Estes, 1989; Xia et al., 2017).

Therefore, the non-specificity of AVG enzymatic inhibition allows us to explore the effects of N metabolism impairment downstream GS/GOGAT cycle assimilation in order to investigate the fundamental role of aminotransferases network on the root morphogenetic program and root nutrient ion uptake through the AA content imbalance and structure-function analyses (Le Deunff and Lecourt, 2016; Le Deunff et al., 2016). This study investigated to what extent root architecture modifications induced by high concentrations of AVG treatments (>5 μM) are solely attributed to ethylene and IAA biosynthesis inhibition. It examined whether the elongation changes of exploratory root systems induced by AVG affect nitrate uptake and N metabolism. Changes in ^15^N absorption and allocation in the plantlets were achieved using differential labeling of K^15^NO_3_ and ^15^Nglutamate. Quantitative analyses of AA profiles using Ultra Performance Liquid Chromatography (UPLC) reveal significant changes in N metabolism induced by AVG treatment and the restoration effect caused by AVG + Glu treatment. Overall, the results demonstrate that changes in the root morphogenetic program induced by AVG treatment are associated with an alteration of N status and histidine homeostasis and a remodelling of N and C metabolism through root aminotransferases network.

## Materials And Methods

### Plant Material and Growth Conditions

The oil seed rape *(Brassica napus* L.) seeds used in this study were the winter oil seed rape cultivar Capitol. The seeds were treated with 70% alcohol for 3 min, followed by three rinses in sterile water and placed on imbibed filter paper (Watmann 3M) saturated with sterile water within Petri dishes (12 × 12 cm) for 48 h in the dark at room temperature. The germinated seeds were then selected according to their radicle length (5–6 mm) and 4 plantlets were transferred onto new Petri dishes (12 × 12 cm), filled with 50 ml of solidified agar culture medium with different chemical treatments. Basic medium contained 0.4 mM KH_2_PO_4_, 0.15 mM K_2_HPO_4_, 1 mM K_2_S0_4_, 0.5 mM MgSO_4_, 3 mM CaCl_2_, 0.2 mM Fe-Na EDTA, 14 μM H_3_B0_3_, 5 μM MnSO_4_, 3μM ZnSO_4_, 0.7 μM CuSO_4_, 0.7 μM (NH_4_)_6_ Mo_7_O_24_ and 0.1 μM CoCl_2_ and solidified with 0.8% (W/V) agar (Sigma-Aldrich, A-7002), pH 6.75. This basic medium was supplemented with 1 mM KNO_3_ as a sole nitrogen source for all the treatments. Ethylene biosynthesis inhibitor AVG (aminoethoxyvinylglycine, sigma-aldrich, A 6685) was dissolved in sterile water to final stock concentration of 10 mM. Adequate volumes of chemicals were added to 50 ml autoclaved cultured medium with or without 1 mM Glutamate in a falcon tube, mixed and then poured into Petri dishes under laminar sterile air flux. After addition of two-day-old germinated plantlets, Petri dishes were half sealed with adhesive tape. The dishes were placed vertically in a growth chamber at 22°C under 16/8 light/dark regime with a light intensity of 200 μmol. m^-2^ s^-1^ and 70% relative humidity.

### Morphometric Analyses of Root System Length and Cotyledon Surface Area

Different concentrations of AVG (0.5; 1; 5; 10; 20 μM) or treatments with 10 μM AVG and 10 μM AVG + 1 mM glutamate in the presence of 1 mM KNO3 were applied to the seedlings during 5 d as described by Leblanc et al., 2008. 

Effects of treatments on the exploratory root system (primary and lateral roots) and shoot area were measured every day during a kinetic of 5 days or after 120 h of treatment. For each treatment four repeats corresponding to four different agar plates comprising 4 plantlets were harvested. The root and shoot parts of the plantlets were excised. Plantlet roots were washed in 1 mM CaSO_4_ solution for 1 min at room temperature before to be placed in demineralized water solution and analyzed by WinRHIZO scan system. Shoots were placed on transparent slide and photocopied (copier Kyocera Mita, KM-2030) to further quantify the total leaf area by WinRHIZO scan system (Regent Instruments inc., Quebec, Canada). After these treatments, the roots and the shoots were dried on tissue paper and placed into 2 ml Eppendorf tubes. The tubes were weighed to get root and shoot fresh weight. Then, tubes were dried in oven for 72 h at 60°C and root and shoot parts were weighed to get their dry weight. Before isotope analyses, the shoots and roots were ground separately for 2 min to fine powder with inox beads of 0.4 mm diameter in oscillating grinder (Retsch mixer mill, MM301).

### Net K^15^NO_3_^-^ Uptake and Isotope Analysis

Net uptake of NO_3_^-^ and glutamate was obtained by ^15^N labeling. For each point of the time-course study (24, 48, 72, 96 and 120 h) the medium was supplemented with K^15^NO_3_ or K ^15^NGlutamate (atom% ^15^N: 1%). The total ^15^N amount was determined for roots and shoots. Then, the ^15^N absorption and translocation were calculated as ^15^N μg .cm-1 root length and ^15^N μg shoot/(^15^Nμg shoot + ^15^Nμg root)*100, respectively. The ^15^N analyses were performed using an analyser (EA 3000, Eurovector, Milan Italy) coupled with a isotopic mass spectrometer (isoprime X, GV instrument, Manchester, UK).

### Amino Acids Profiling

Amino acid profiling was performed on shoot and root material using the ACQUITY Ultra Performance LC (UPLC) separation system (Waters corp., Milford, USA). Plant tissues were collected, freeze-dried and homogenized with a 4-mm steel ball for 1 min at 30/s frequency. Mehtanol-chloroform-water-based extractions were made on 10 mg of the resulting dry powder. The powder was suspended in 400 µl of Mehtanol containing 200 µM DL-3-aminobutyric acid (BABA) as an internal standard and agitated at 1,500 rpm for 15 min at room temperature. 200 µl of chloroform was then added and samples were agitated at 1,500 rpm for 5 min at room temperature. Finally, 400 µl of ultra-pure water was added and samples were vigorously mixed and then centrifuged at 13,000 g for 5 min at 4°C. The upper phase containing AA was transferred to a clean microtube, dried under vacuum and the dry residue was resuspended in 600 µl of ultra-pure water. A 5 µl aliquot of the resulting extract was used for derivatization according to the AccQ•Tag Ultra Derivatization Kit protocol (Waters corp., Milford, USA) and then derivatized AA were analysed using an ACQUITY UPLC^®^ system (Waters corp., Milford, USA). One µl of the reaction mix was injected onto an ACQUITY UPLC BEH C18 1.7 µm 2.1 × 100 mm column heated at 55°C. Elution of AA was performed with a mix of 10-fold diluted AccQ•Tag Ultra Eluent (A) and acetonitrile (B) at 0.7 ml.min^-1^ flow according to the following gradient: initial, 99.9% A; 0.54 min, 99.9% A; 6.50 min, 90.9% A, curve 7; 8.50 min, 78.8% A, curve 6; 8.90 min, 40.4% A, curve 6; 9.50 min, 40.4% A, curve 6; 9.60 min, 99.9% A, curve 6; 10.10 min, 99.9% A. AA were detected at 260 nm using a photo diode array detector and were subsequently identified and quantified with the individual external standard calibration curves.

## Results

### Chronic Treatments With Increasing AVG Concentrations Reduce the Root Elongation Without Altering the Shoot Growth

**Figure 1:**
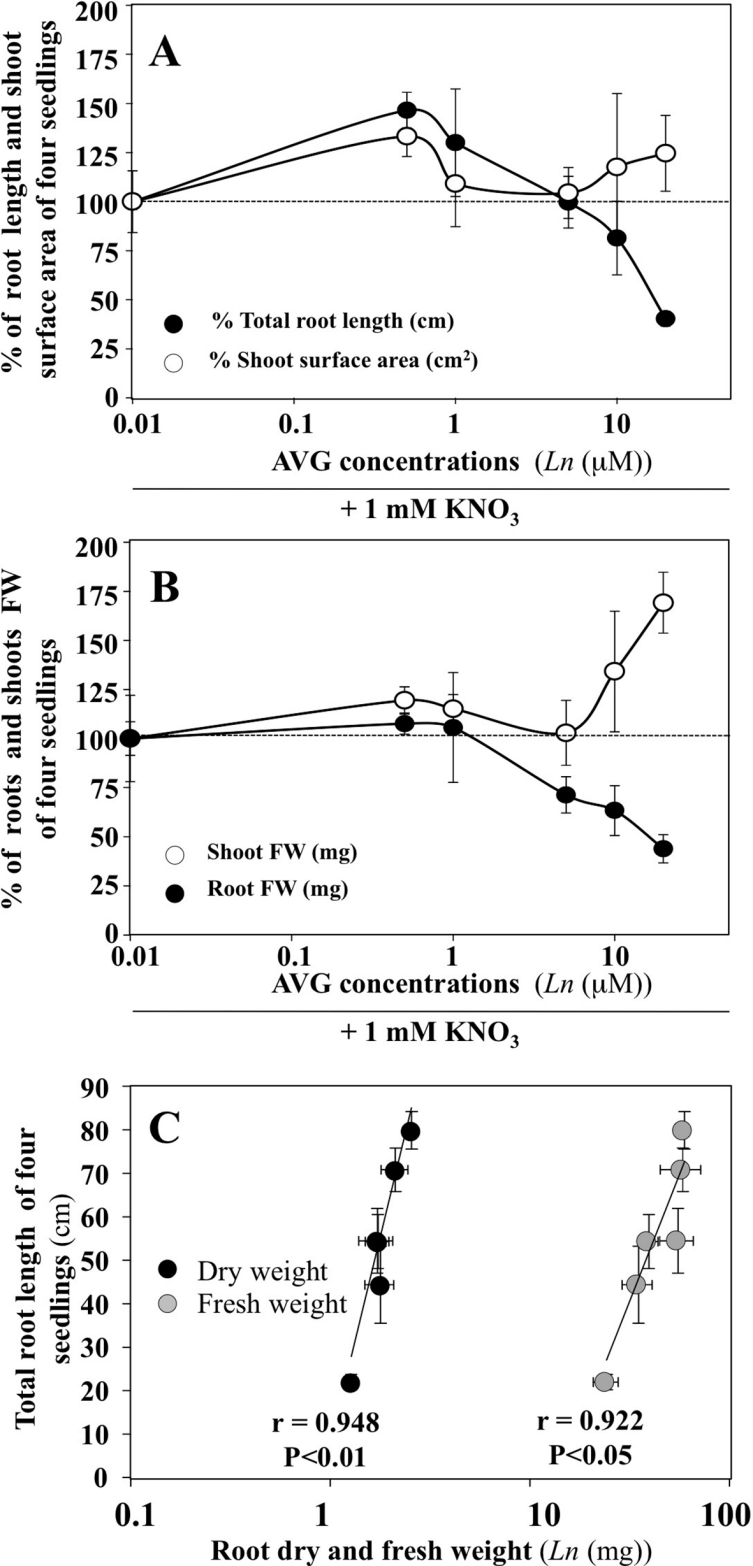
Impacts of AVG treatments on growth of *Brassica napus* seedlings treated over 5 days on agar plates under homogeneous feeding of 1 mM KNO_3_. **(A)** Dose-response curves of AVG treatments on % of total root length and shoot surface area compared to control seedlings. **(B)** Dose-response curves of AVG treatments on % of shoot and root FW compared to control seedlings. **(C)** Correlation between total root length and root dry and fresh weight. Values are the average ( ± SE) of 4 agar plates (N 4) of four seedlings each. Significant differences between control (1mM KNO_3_) and treatments are given for *p < 0.05; ***p < 0.005; (t-test).

Changes in the exploratory root system elongation (primary and lateral roots) and shoot surface area expansion in *B. napus* plantlets were examined on vertical agar plates under a homogeneous supply of 1 mM nitrate with different concentrations of AVG (0.5 to 20 μM) after 120 h of treatment (**Figure 1**). AVG-treated plantlets exhibited a biphasic dose-response curve with an increase in root elongation below 1 μM followed by a sharp reduction in exploratory root system development compared to control plantlets AVG-untreated (**Figure 1A**). Roots grown with 10 and 20 μM AVG reduced roughly 18.6% and 59.7% their root elongation compared to roots grown without AVG. The dose-dependent reduction in the elongation of exploratory root system was accompanied by a progressive and significant reduction in root fresh and dry weights (f.wt and d.wt) as indicated by high linear correlations obtained between root length and root f.wt and d.wt (**Figures 1B, C**). Although AVG concentrations did not significantly affect shoot surface expansion, AVG induced a significant increase in leaf biomass resulting in an increase in leaf specific mass (weight per cm^2^, data not shown) as previously observed (Leblanc et al., 2008). Therefore, the differential morphological effects measured between shoot and root raised the question: does AVG treatment induce a profound remodeling of N and C metabolism in the roots and shoots irrespective of the inhibition of ethylene and IAA biosynthesis?

### Root Elongation Is Significantly Inhibited by AVG Treatment But Partially Restored by 1 Mm L-Glutamate Treatment

**Figure 2:**
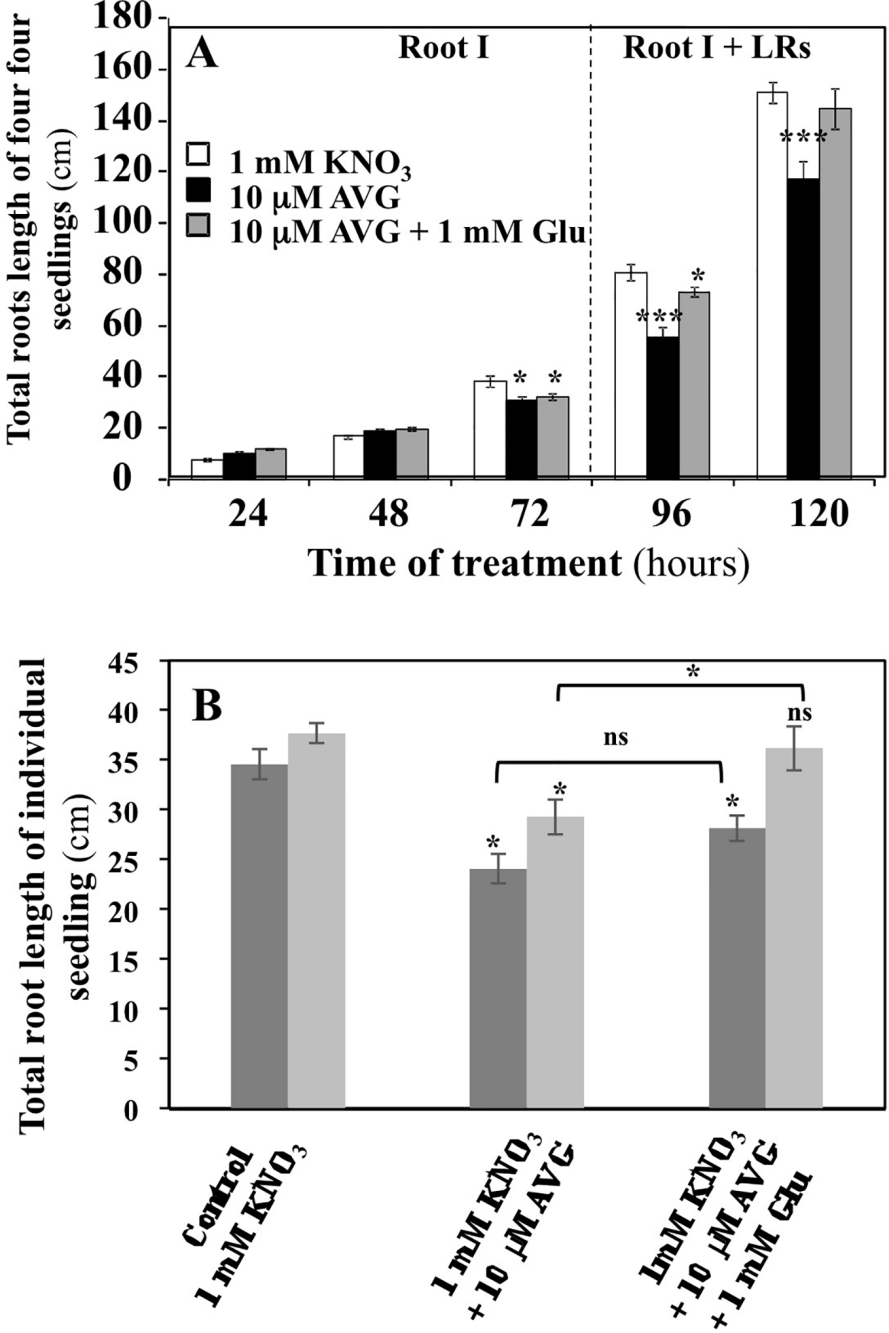
Effects of chronic treatment by L-glutamate on the restoration of root length in AVG-treated seedlings growing over 5 days on agar plates under homogeneous feeding of 1 mM KNO_3_. **(A)** Restoration of root length induced by 1 mM glutamate treatment in AVG-treated seedlings during time course of experiment. Values are the average ( ± SE) of 4 agar plates (n = 4) of four seedlings each. **(B)** Partial restoration of seedlings root length induced by 1 mM glutamate treatment on 10 μM AVG-treated seedlings after 120 h of treatment between two different and independent experiments. Values are the average ( ± SE) of 11–16 plantlets. Significant differences between control (1 mM KNO_3_) and treatments are given for *p < 0.05 (t-test).

Dynamics of the inhibition of the exploratory root system elongation by 10 μM AVG treatment were followed during the time course of experiment (**Figure 2A**). Root growth inhibition by AVG became significant only after 72 h of treatment. Until 72 h, the plantlets may have heterotrophic growth and develop only a primary root from their cotyledon lipid reserves. Then, the lateral roots (LRs) appear with the greening of cotyledons and the beginning of autotrophic growth of the plantlets (**Figure 2A**; Marek and Stewart, 1992). Co-treatment of the plantlets with 10 µM AVG + 1 mM Glu, a major amino acid in N assimilation, induced a restoration of the length and biomass of exploratory root system after 72 h of treatment (**Figures 2A, B**). Glutamate was chosen because of its central position in N and AA metabolism in plant after N assimilation by the *GS/GOGAT* cycle. This partial morphological recovery indicates that the AVG inhibition spectrum is likely to extend beyond enzymes involved in biosynthesis of ethylene and IAA such as *ACS* and *TAA* (**Figures 2A, B**), while other PLP-dependent enzymes of α family involved in N metabolism are also the potential targets of AVG inhibition (Berkowitz et al., 2006).

### AVG Treatment Increases K^15^NO_3_ Uptake Whereas AVG + K^15^Nglutamate Treatment Inhibits K^15^NO_3_ Uptake and Increases ^15^Nglutamate Uptake

**Figure 3:**
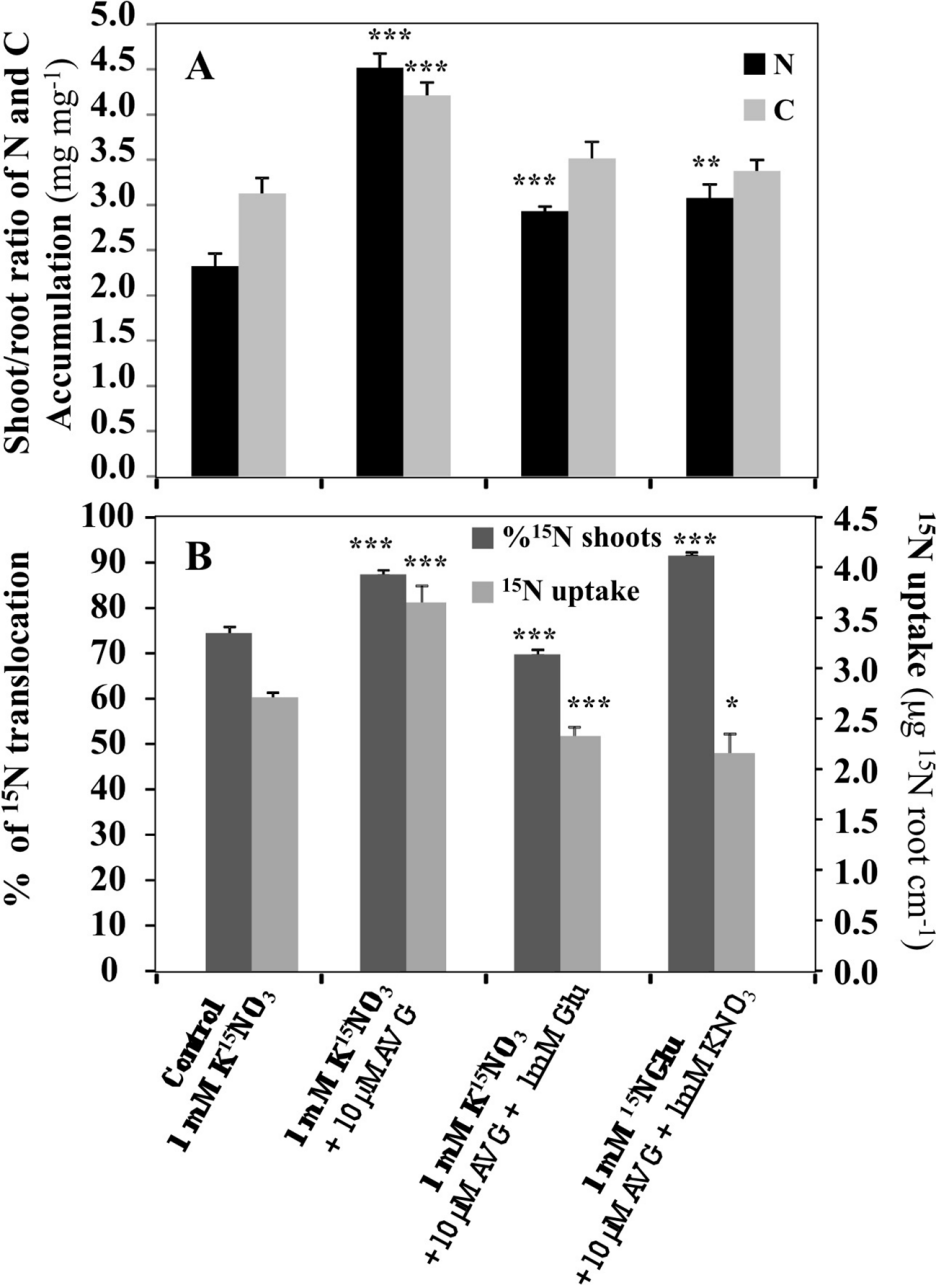
Comparison of AVG and AVG + Glu treatments on the shoot/root ratio of total amounts of nitrogen and carbon **(A)** and ^15^N uptake and translocation **(B)** in *Brassica napus* seedlings treated over 5 days on agar plate with 1 mM KNO_3_ (control). ^15^N uptake was measured either by treating the seedlings with K^15^NO_3_ or ^15^NGlu. Values are the average ( ± SE) of 4 agar plates (n = 4) of four seedlings each. Significant differences between control and treatments are given for *p < 0.05; **p < 0.01; ***p < 0.005; (t-test).

In order to better characterize the root morphological inhibition and restoration effects induced by AVG and AVG + Glu treatments in relation with N uptake and allocation, a differential ^15^N labeling of nitrate (K^15^NO_3_) and glutamate (K^15^NGlu) was used to examine ^15^N absorption and allocation issue from nitrate or glutamate in the plantlets after 120 h of treatment (**Figure 3**). ^15^NO_3_^-^ accumulation revealed that AVG treatment induces a significant increase in ^15^NO_3_ uptake and ^15^N translocation to the shoots (**Figure 3B**). Accordingly, compared to control (KNO_3_) in KNO_3_ + AVG treatment the shoot/root ratios of N and C contents were significantly increased (**Figure 3A**). By contrast, in co-treated plantlets with 1 mM K^15^NO_3_ + 10 μM AVG + 1 mM Glu, ^15^NO_3_ uptake and ^15^N translocation were significantly decreased compared to control whereas in 1 mM KNO_3_ + 10 μM AVG + 1 mM K^15^NGlu, ^15^NGlu uptake and ^15^N translocation were significantly increased (**Figure 3B**). The high ^15^N accumulation in the shoots (90%) in plantlets treated with AVG + 1 mM K^15^NGlu indicated that the ^15^NGlu taken up by the roots was rapidly translocated to the shoots for assimilation and/or storage. This result is consistent with the compensation mechanism between KNO_3_ and KNO_3_ + Glu uptake observed in a previous work (Leblanc et al., 2013). Indeed, when Glu availability increases, ^15^NGlu is strongly taken up and translocated to the shoots whereas nitrate uptake is progressively inhibited (Leblanc et al., 2013). This ensures the maintenance of a high N status of the plant under a mineral or organic N supply. Since Glu treatment also inhibits nitrogen assimilation in roots and shoots (Leblanc et al., 2013), this suggests that a qualitative reprogramming of N metabolism is induced during an organic N supply like Glu. To highlight the effects of AVG and AVG + Glu treatment on changes in the N metabolism and root and shoot growth, changes in total free AA contents were determined after 5 days of treatment by ultra high-performance liquid chromatography (UPLC) analysis.

### Inhibition of the Aminotransferase Network by AVG Treatment Creates a Physiological Situation of N Limitation

**Figure 4:**
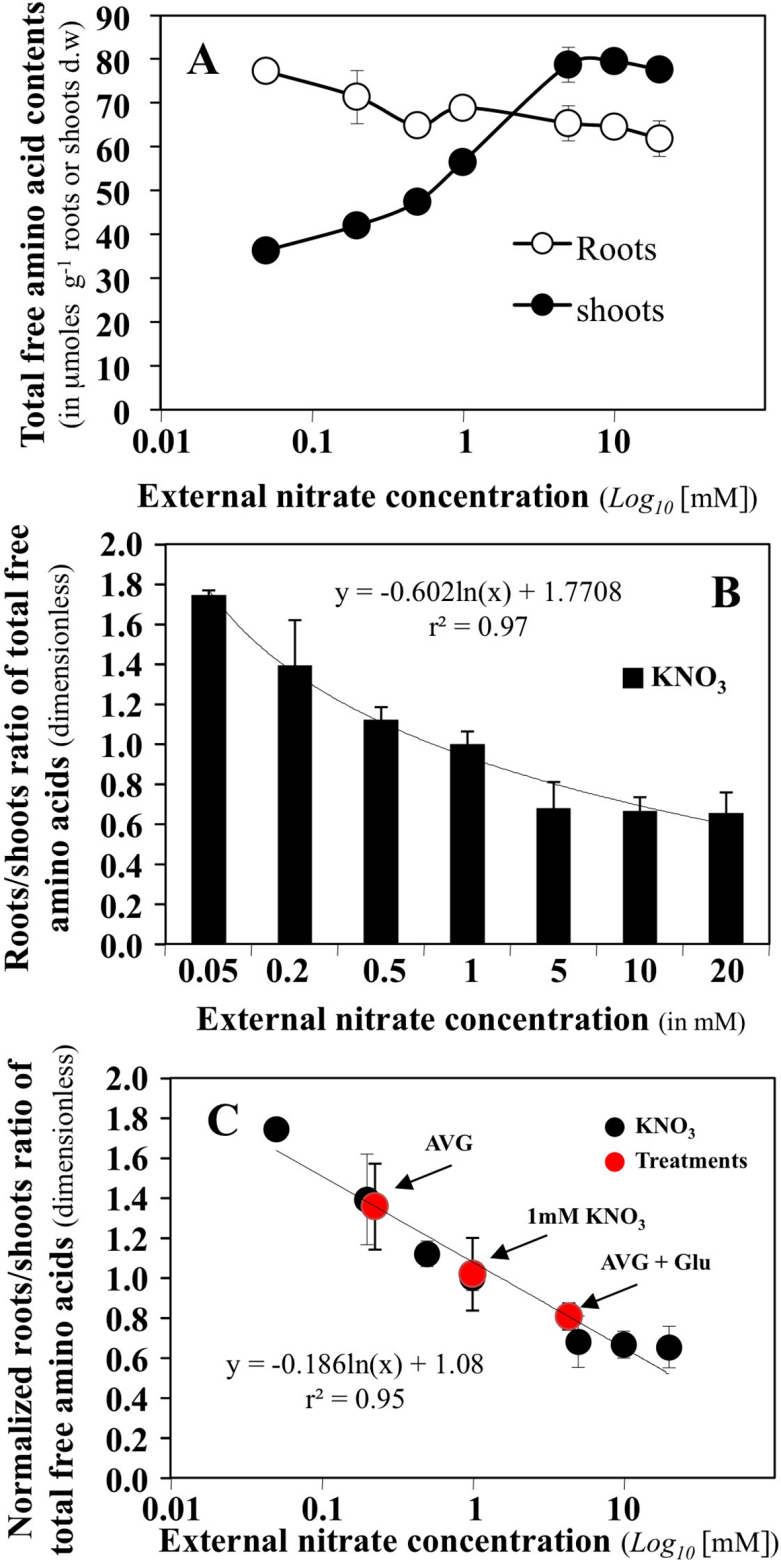
Determination of a synthetic N status parameter from variations of total amino acid contents in the roots and shoots of *Brassica napus* seedlings growing over 5 days on agar plates under homogeneous feeding of nitrate. **(A)** Changes in total free amino acid contents in the roots and shoots of *B. napus* seedlings treated with different external nitrate concentrations. **(B)** Roots/shoots ratio of total free amino acids defined as a synthetic parameter of N status of *B. napus* seedlings. **(C)** Normalized N status parameter from 1 mM KNO_3_ treatment and estimation of the values of this parameter for seedlings treated with 10 μM AVG + 1 mM KNO_3_ and 10 μM AVG + 1mM Glu + 1 μM KNO_3_.Values are the average ( ± SE) of 3 agar plates (n = 3) of four seedlings each.

The effect of a high range of external concentrations in nitrate on the content of total free amino acids in the roots and shoots of *B. napus* seedlings growing in agar plates has been previously determined (Le Ny et al., 2013). Amino acids are the first products of nitrate assimilation and varied in the roots and shoots depending on nitrate supply and the plant N status (**Figure 4A**). Low N availability, decreased the translocation of ^15^N to the shoot compartment and was associated with a depletion of AA levels in shoot tissues, while AA levels were maintained or increased in the root. Conversely, when increasing nitrate availability, the restoration of high ^15^N translocation to the shoot was associated with a two-fold increase in AA levels in the shoot and a decrease in the root (Le Ny et al., 2013). So, we used the root/shoot ratio of total free amino acids (**Figure 4B**) to define a synthetic parameter of global N status of *B. napus* seedlings in a steady-state growth under homogeneous feeding of nitrate. This parameter allows characterising the effect of AVG and AVG + Glu treatments on the plant N status through aminotransferase network activities in relation with nitrate availability (**Figure 4C**). Thus, high ratio values indicate a physiological situation corresponding to a N limitation with an increase in total AA in the root and a decrease in the shoot (**Figures 4A, B**). As shown in **Figure 4C**, treatment with AVG induces a near-deprivation nitrogen limitation condition equivalent to 0.2 mM KNO_3_ availability. This corresponds to a 5-fold reduction in the nitrate concentration perceived by the roots (**Figure 4C**) while AVG + Glu treatment induced a near-excess nitrogen condition equivalent to 4.8 mM KNO_3_ availability (**Figure 4C**). To highlight the qualitative and quantitative changes of N metabolism induced by AVG and AVG + Glu treatment, the individual free AA contents of the roots and shoots were then analysed.

### The Nitrogen Assimilation Pathway is More Impaired by AVG Treatment in the Root Than in the Shoot

**Figure 5:**
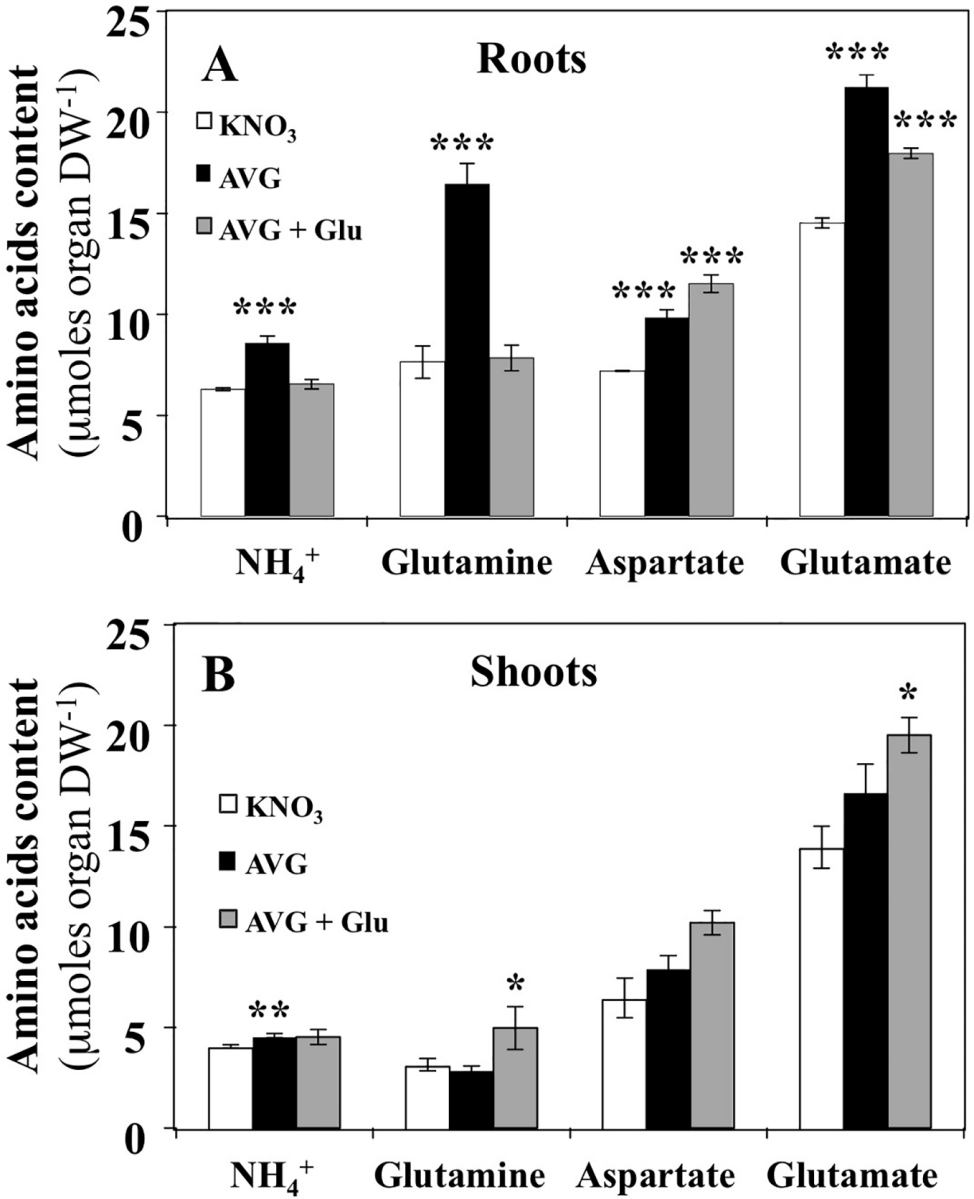
Changes in NH_4_^+^ and free amino acids contents induced by 10 μM AVG and 10 μM AVG + 1 mM Glu treatments in the root and shoot tissues of *Brassica napus* seedlings treated over 5 days on agar plates under homogeneous feeding of 1 mM KNO_3_. **(A)** Changes of NH_4_^+^, glutamine, aspartate and glutamate contents in the root tissues. **(B)** Changes of NH_4_^+^, glutamine, aspartate and glutamate contents in the shoot tissues. Values are the average ( ± SE) of 3 agar plates (n = 3) of four seedlings each. Significant differences between control (1mM KNO_3_) and treatments are given for *p < 0.05; **p < 0.01; ***p < 0.001; (t-test).

The effect of AVG treatment on the content of individual free AA was more severe in roots than in shoots (**Figure 5**). In roots, AVG treatment induced a significant increase of the three major AA: glutamine, aspartate and glutamate (**Figure 5A**). Since glutamine synthase (*GS*) and glutamate synthase (*GOGAT*) are not PLP-dependent enzymes, the increase in glutamine, glutamate and NH_4_^+^ contents in root tissues must be caused by inhibition in the protein synthesis or non-specific inhibition of some aminotransferases belonging to the aminotransferases network downstream *GS/GOGAT* cycle. Likewise, the restoration of glutamine and NH_4_^+^ concentrations to control levels following glutamate treatment can be explained by the reduction of nitrate uptake rate that in turn induces a decrease in NH_4_^+^ and glutamine production by *GS/GOGAT* cycle (**Figures 3B**, **5B**). In addition, the accumulation of significantly higher levels of glutamate is caused to higher absorption of K^15^NGlu during AVG + Glu treatment (**Figures 3B**, **5A**). The observed increase in aspartate levels could result from a differential inhibition of both types of *AAT* by AVG (Maeda et al., 2011; Maeda and Dudareva, 2012; de la Torre et al., 2014). Indeed, the plastidic (p) Eukaryotic Type (*ET-pAAT*) and Prokaryotic Type (*PT-pAAT*) of *AAT* belong to the subfamily Iα and Iβ respectively and present only 15% identity in the protein sequence between their members (Jensen and Gu, 1996). Alternatively, inhibition by AVG of the cystathionine β-lyase (CbL) and *ACS* activities may explain decrease in the use of Asp and increase in the root Asp levels (Clausen et al., 1997; Ravanel et al., 1998). Likewise, cellular compartmentation of *AAT* isoenzymes localised within cytosolic, plastidial, peroxisomal and mitochondrial compartments would explain aspartate accumulation in AVG-treated plantlets and the lack of recovery after AVG + Glu treatment (**Figure 5A**).

### Variations Induced by AVG and AVG + Glu Treatments on the Precursor Levels of Ethylene and Auxins

**Figure 6:**
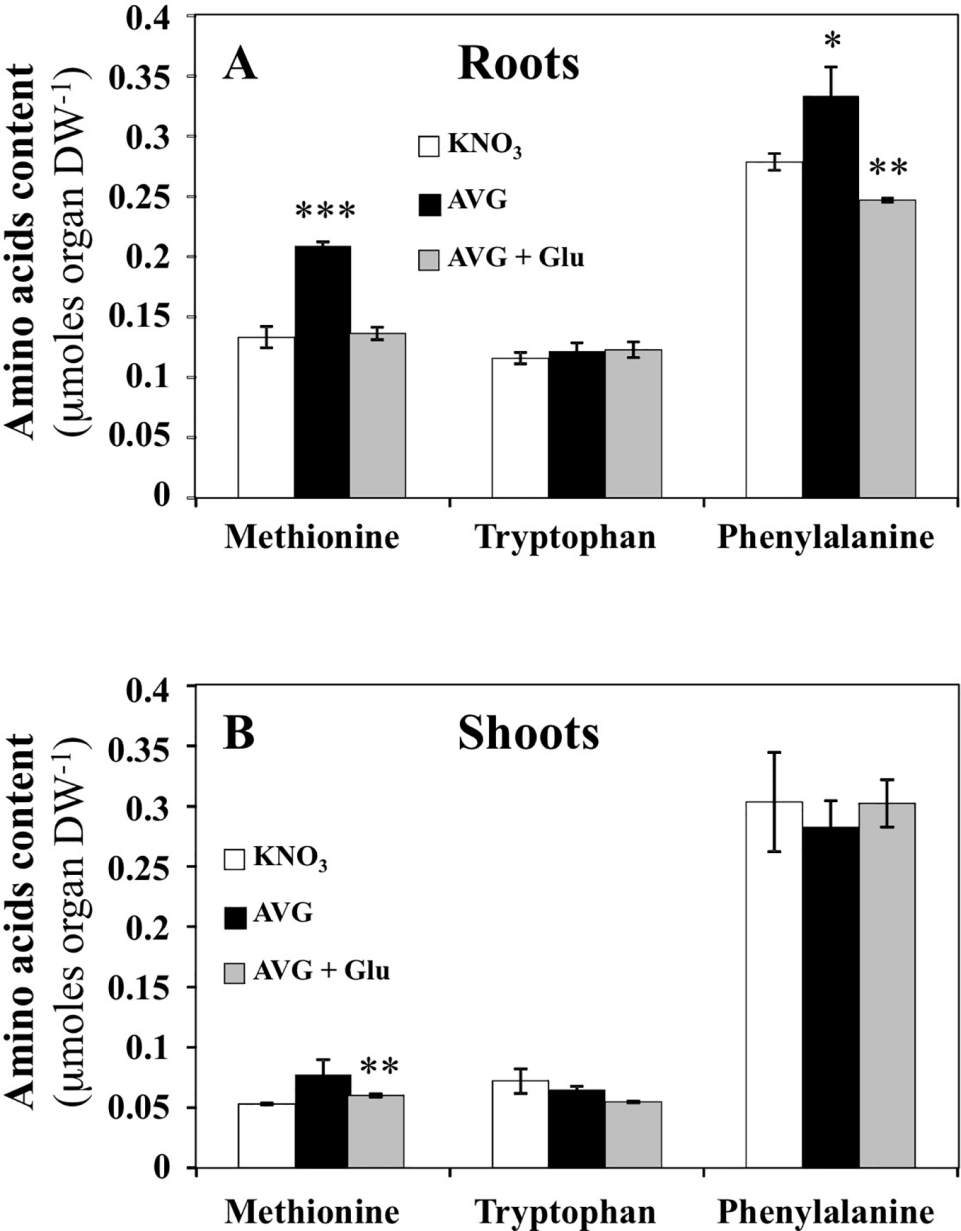
Changes in free amino acids contents induced by 10 μM AVG and 10 μM AVG + 1 mM Glu treatments in the root and shoot tissues of *Brassica napus* seedlings treated over 5 days on agar plates under homogeneous feeding of 1 mM KNO_3_. **(A)** Changes of methionine, tryptophan and phenylalanine contents in the root tissues. **(B)** Changes of methionine, tryptophan and phenylalanine contents in the shoot tissues. Values are the average ( ± SE) of 3 agar plates (n = 3) of four seedlings each. Significant differences between control (1 mM KNO_3_) and treatments are given for *p < 0.09; **p< 0.01; ***p < 0.001; (t-test).

Since ethylene and auxins (IAA and PAA) play a central role in root growth and development (Muday et al., 2012; Sugawara et al., 2015; Le Deunff, 2018), we checked whereas significant changes in contents of their precursors methionine, tryptophan and phenylalanine respectively, could explain the observed differences in root elongation between treatments (**Figure 6**). Indeed, *ACS* and *TAA1/TARs* are cytosolic aminotransferases targeted by AVG, Rhizobitoxine and AOA inhibitors (Lieberman et al., 1974; Hanson and Kende, 1976; Berkowitz et al., 2006; Soeno et al., 2010). *ACS* is a PLP-dependent enzyme that catalyzes the irreversible conversion of S-adenosyl-methionine into 1-aminocyclopropane-1-carboxylic acid (ACC, ethylene precursor) and 5-methylthio-adenosine. *TAA1/TARs* are PLP-dependent enzymes essential in indol-3-pyruvic acid (IPA) branch of the auxins biosynthetic pathway (Stepanova et al., 2008; Tao et al., 2008). They convert tryptophan and phenylalanine with α-ketoglutarate or pyruvate to indol-3-pyruvic acid and phenylpyruvic acid corresponding respectively to the precursors of IAA and PAA (Sugawara et al., 2015). In roots of AVG-treated plantlets, methionine and phenylalanine contents were significantly increased (**Figure 6A**), while in shoots only methionine showed slight and non-significant accumulation (**Figure 6B**). A recovery of methionine and a significant decrease in phenylalanine contents in roots was induced by AVG + Glu treatment (**Figure 6A**). However, tryptophan concentrations were unchanged in the roots and shoots regardless of the treatment used (**Figures 6A, B**). To highlight the quantitative changes in the levels of methionine, tryptophan and phenylalanine with respect to the nitrogen metabolism and shoot and root morphological changes, the AA levels induced by the treatments were repositioned on the responses curves obtained across a whole range of external nitrate concentrations.

### Changes in Trp Levels Are significantly Correlated With Morphological Changes and Nitrogen or Carbon Accumulation in the Shoots and Roots

**Figure 7:**
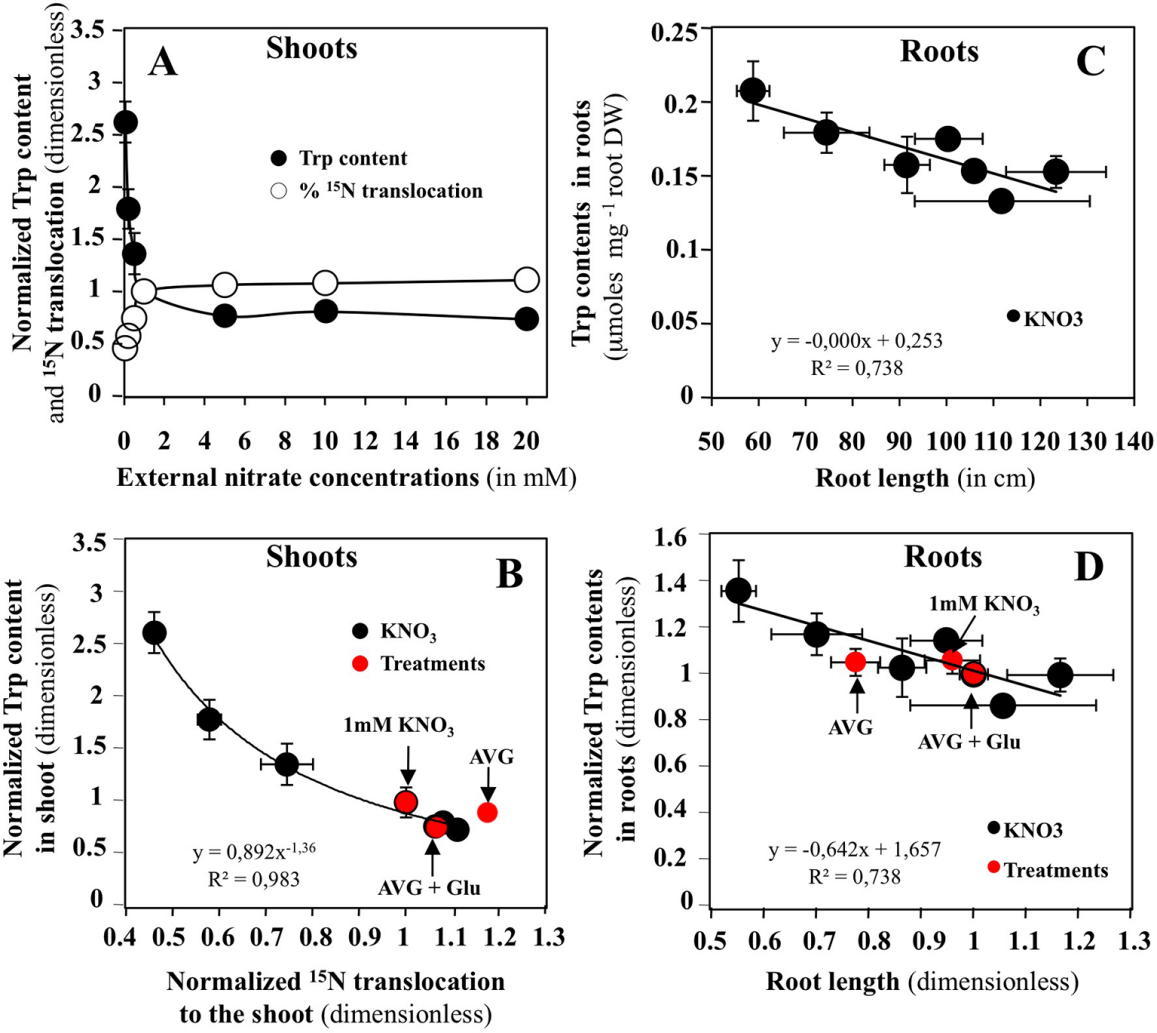
Effects of nitrate on different roots and shoots bichemical and morphological variables in *Brassica napus* seedlings treated with varying external nitrate concentrations for 5 days on agar plates. **(A)** Responses of Trp content and % of ^15^N translocation in shoots to increasing nitrate concentrations. **(B)** Relationship between the shoots Trp content and % of ^15^N translocation after normalization of the values relative to 1 mM KNO_3_ (control). **(C)** Relationship between the roots Trp content and the root length. **(D)** Same relationship as in C but the values have been normalized relative to 1 mM KNO_3_ (control). Values are the average ( ± SE) of four repeats of four seedlings each. Red points correspond to estimation of the values of same variables in another experiment for seedlings treated with 1 mM KNO_3,_ 10 μM AVG + 1 mM KNO_3_ and 10 μM AVG + 1mM Glu + 1 mM KNO_3_.

Relationships between changes in Met, Trp and Phe levels with common indicators of root and shoot growth, such as the N translocation and accumulation, shoot surface area, root length, fresh and dry weights in response to a whole range of external nitrate concentrations have been systematically sought from a previous study (Le Ny et al., 2013). Significant correlations with shoot and root growth changes were only found for changes in Trp levels (**Figures 7** and **S1**). **Figure 7** showed that changes in Trp contents in the shoots are significantly correlated with ^15^N translocation (**Figures 7A, B**) and ^15^N accumulation (**Figures S1**). The same is not true for the roots where changes in Trp content are mainly related to variations in root DW and length (**Figures 7C, D** and **S1**). After normalization of all the graphs relative to 1 mM KNO_3_ treatment, the values obtained after treatments with 10 μM AVG + 1 mM KNO_3_ and 10 μM AVG + 1 mM Glu + 1 mM KNO_3_ were positioned on the N response curves.

In shoots, the results confirm AVG treatment is associated with an increase in nitrate uptake and translocation rates (**Figure 7B**). Intriguingly, AVG induced a translocation rate of N greater than 87% (**Figure 7B**). Such a translocation rate is only found in seedlings treated with more than 20 mM nitrate. Indeed, the control seedlings treated with 1 mM KNO_3_ have a translocation rate of 75%. Even AVG + Glu treatment fails to induce such a level of translocation rate (87%). AVG treatment leads to a decrease in Trp content of the shoots up to a 2 mM KNO_3_ treatment, whereas the Trp level induced by AVG + Glu treatment is equivalent to approximately 6 mM of treatment with KNO_3_ (**Figure S1B**). Similarly, AVG + Glu treatment increases the amount of ^15^N uptake and translocation provided by nitrate and Glu and provoked a greater decrease in Trp levels in the shoots. Because shoot growth is not impaired by the treatments, the results suggest that in shoots, the alternative IAA biosynthesis pathways are used from Trp to produce IAA necessary for growth. In roots, Trp levels were mainly correlated with root DW and root length (**Figures 7C, D** and **S1**). The results showed that AVG treatment induced a reduction in root length and DW associated with an accumulation of Trp, whereas AVG + Glu treatment resulted in an increased length of the root associated with a decrease in Trp level (**Figures 7C, D**). This leads to the conclusion that the alternative biosynthesis pathways of IAA are not or little used in roots to compensate for AVG inhibition of *TAA* catalytic functions. In order to find other specific determinants induced by AVG in inversion of shoot and root growth compared to treatment with increasing nitrate concentrations, changes in individual levels of the other AAs in roots and shoots have also been analyzed.

### AVG Treatment Impairs the Content in Most Amino Acids in the Roots More Sharply Than in the Shoots

**Figure 8:**
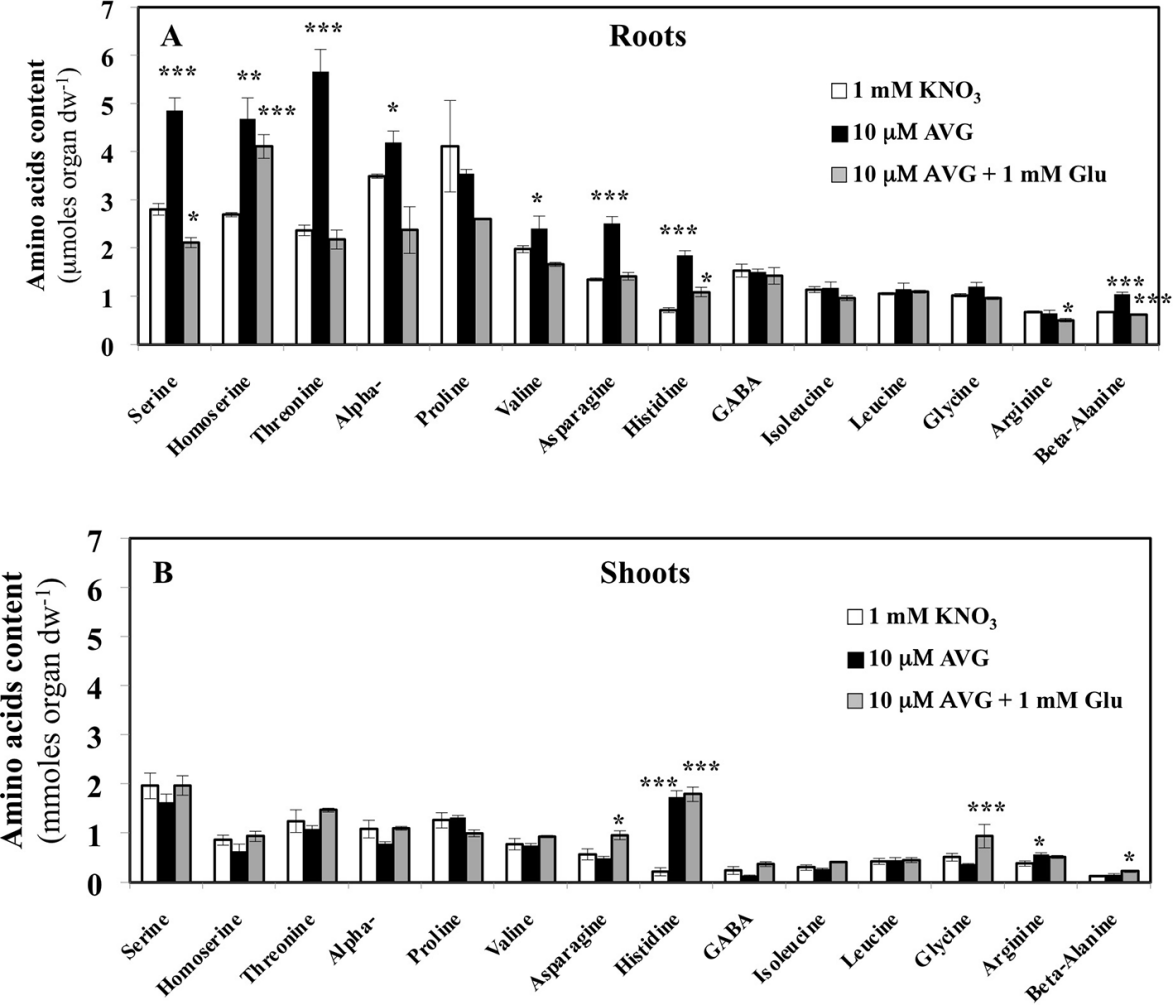
Changes in free amino acid contents induced by 10 μM AVG and 10 μM AVG + 1 mM Glu treatments in the root **(A)** and shoot tissues **(B)** of *Brassica napus* seedlings treated over 5 days on agar plates under homogeneous feeding of 1 mM KNO_3_. Values are the average ( ± SE) of 3 agar plates (n = 3) of four seedlings each. Significant differences between control (1 mM KNO_3_) and treatments are given for *p < 0.05; **p< 0.01; ***p < 0.005; (t-test).

Contents of the other AA showed that roots are more responsive than shoots to AVG treatment (**Figure 8**). In addition, in most cases glutamate treatment induced a recovery of AA in the roots to the control levels (**Figure 8**). In control plantlets (1 mM KNO_3_), total AA content in root tissues was about 1.8-fold higher than total AA content in the shoots (**Figure 8**). This result was in agreement with previous AA profiling obtained with the same *B. napus* line grown in similar conditions (**Figure 4A**, Le Ny et al., 2013). However, relative to external nitrate concentrations, the 1 mM KNO_3_ + 10 μM AVG treatment provoked a quantitative effect on the N metabolism balance between the shoots and roots as previously exemplified in **Figures 4C**. In the root tissues, significant increases in levels of several AA such as serine (Ser), threonine (Thr), α-alanine (Ala), β-alanine (β-Ala), valine (Val), asparagine (Asn) and histidine (His) were observed in AVG-treated plantlets relative to controls (**Figure 8A**). In plants, AA can be grouped depending on whether their carbon skeleton originates from glycolytic, TCA cycle or the oxidative pentose phosphate pathway (**Figure S2**, Coruzzi 2003; Stitt et al., 2002). As most of these AA are the end products of several metabolic pathways (**Figure S1**), this leads to the conclusion that PLP-dependent aminotransferases involved in their biosynthesis were strongly inhibited by root AVG treatment (**Figure 8A**). As the contents of almost all AA increased with AVG treatment, several hypotheses non-mutually exclusive may be proposed for AVG effects: i) reduction of protein synthesis, ii) induction of proteolysis, and iii) inhibition of catabolism of AA in the roots.

**Figure 9:**
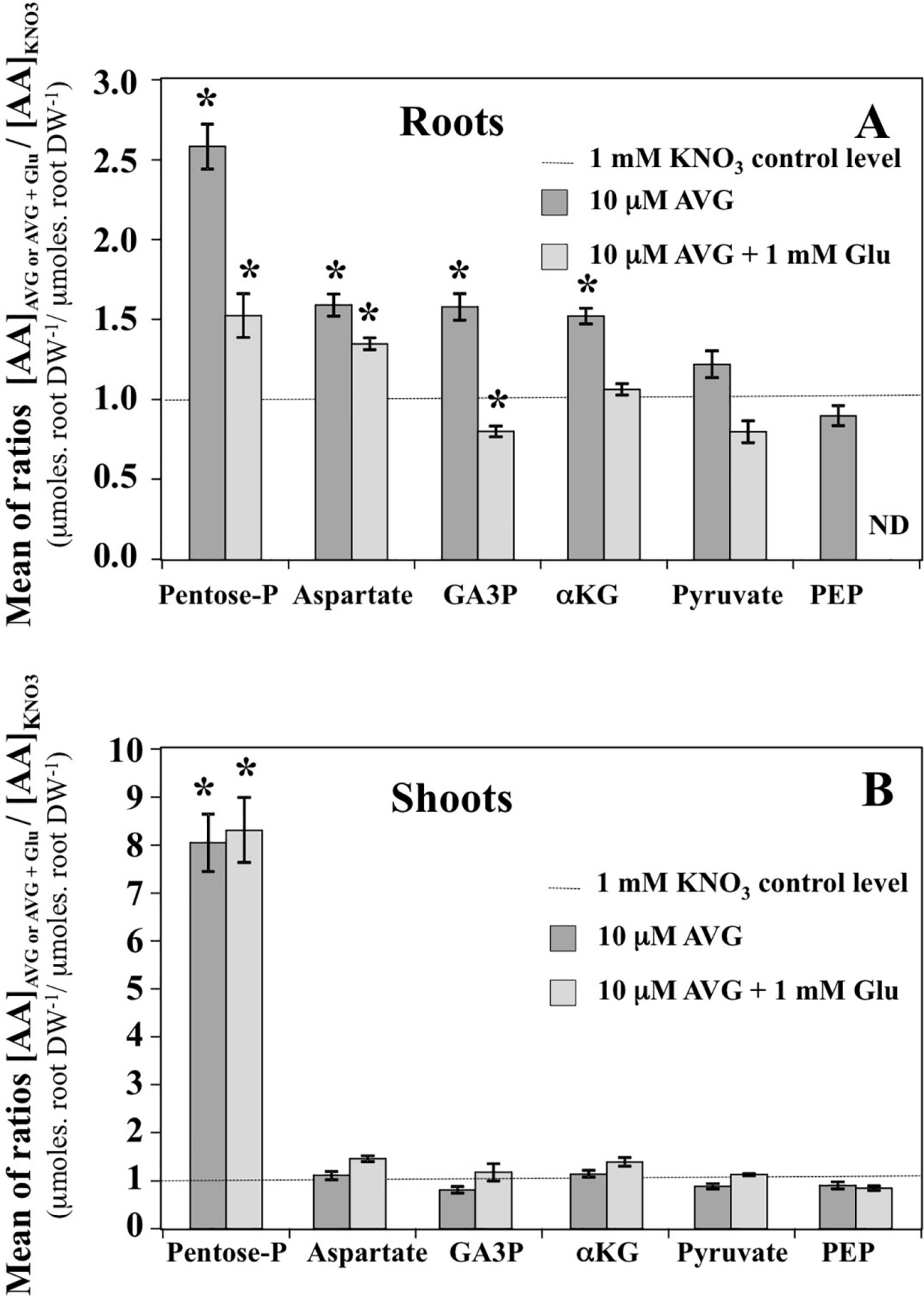
Mean contents of amino acid families in the roots **(A)** and shoots **(B)** tissues of *Brassica napus* seedlings treated with 10 μM AVG or 10 μM AVG + 1 mM Glu relative to those in KNO_3_ treated seedlings (control). Seedlings were growing on agar plates under homogeneous feeding of 1 mM KNO_3_ whatever the treatment. Values are the average ( ± SE) of 3 agar plates (n = 3) of four seedlings each. A significant difference in amino acid ratio is indicated by *p < 0.05 (Benjamini and Hochberg correction for multiple t-test analyses with a false discovery rate of 0.05 between the treatments and KNO_3_ control plants have been done). The composition of the amino acid families is as follows: Pentose-P (His), α-KG (Glu, Gln, Arg, GABA and Pro), Aspartate (Asn, Asp, Thr, Met, Ile and Lys), PEP (Trp, Phe, Tyr), Pyruvate (a-Ala; b-Ala, Val and Leu) and GA3P (Ser and Gly).

In the shoot tissues, the free AA pools were less affected by AVG and AVG + Glu treatments (**Figure 8B**). This can be explained by the lowest AA content in shoots compared to the roots at 1 mM KNO_3_ external concentration (**Figure 4A**), itself further reduced by the AVG-induced N limitation (**Figure 4C**). The overall trend for the three treatments was opposite to the AA patterns found in the root tissues (**Figures 8A, B**). In particular, AA showing a slight decrease in the shoot content induced by AVG treatment were strongly and significantly increased in the roots suggesting a tight coordination of AA levels and recycling between shoots and roots by a N sensing mechanism of N status that remains to be determined. However, AVG and AVG + Glu treatments had no effect on AA levels, except for some AAs such as His, Asp, β-Ala and Gly (**Figures 8B**, **S4**). Amongst amino acid, only His showed a significant increase by 8-fold after AVG treatment and its control level was not restored by glutamate (**Figure 8B**). Further, relative changes in AA levels belonging to the same metabolic pathways compared to control plantlets confirmed conclusions from changes of individuals AA in the roots (**Figure 9A**) and shoots (**Figure 9B**). Repetition of the experiment gave similar results regarding changes in AA contents taken individually (**Figures S3**, **S4**) or grouped by family (**Figure S5**). Only levels of pentose-P family (His) in shoots were lower in this second experiment for both treatments but still significantly different (**Figure S5**).

## Discussion

Although the use of AVG inhibitor is generally associated to ethylene and IAA biosynthesis inhibition in relation with root development (Rahman et al, 2000; Le et al., 2001; Stepanova et al., 2007; Swarup et al., 2007; Růžička et al., 2007), the results demonstrate that the non-specificity of AVG inhibitor creates a physiological situation of N limitation associated with a profound remodelling of AA metabolism that impairs regulation of nitrate uptake and translocation, His homeostasis and root and shoot growth.

### AVG and AVG + Glu Treatments Impair N Metabolism and Challenges the Canonical Regulation of Nitrate Uptake Rate by NH_4_^+^ and Glutamine

Despite an average 22.5% decrease in the exploratory root system, 10 μM AVG-treated plantlets accumulated as much as ^15^N as KNO_3_ control plantlets after 120 h of treatment. This finding confirms earlier data that seedlings treated with 10 μM AVG, which only have three-quarter to half of their exploratory root system and few root hairs, accumulated as much as ^15^N as the control seedlings (Leblanc et al., 2008; Le Deunff et al., 2016). Accordingly, relative to the total root length, nitrate uptake rate was significantly increased in AVG-treated plantlets. As previously demonstrated, the increase in nitrate uptake rate was accompanied by an over-expression of *BnNRT2.1* nitrate transporter gene, the transcription level of which adapts to changes in the roots’ absorbing surface (Leblanc et al., 2008; Le Ny et al., 2013; Lemaire et al., 2013). The up-regulation of nitrate uptake can be explained by AVG induction of a physiological condition similar to a N limitation (low N status) as indicated by a high value of root/shoot ratio of total free amino acids. Indeed, N deprivation conditions is known to induce an up-regulation of NRT2.1 nitrate transporter expression (Lejay et al., 1999; Beuve et al., 2004). Interestingly, the synthetic N status parameter used allows to demonstrate that AA are not directly involved in feed-back regulation of nitrate uptake and nitrate transporters expression as previously supposed (Cooper and Clarkson, 1989; Muller and Touraine, 1992; Krapp et al., 1998; Tillard et al., 1998; Vidmar et al., 2000; Gansel et al., 2001; Beuve et al., 2004). Indeed, it is generally accepted that increase contents of NH_4_^+^ and glutamine down regulated ^15^NO_3_^-^ influx rate and *NRT2.1* expression (Vidmar et al., 2000; Nazoa et al., 2003; Beuve et al., 2004). In AVG-treated plantlets, the endogenous contents of NH_4_^+^, glutamine and glutamate were 2.6-, 1.6- and 1.5-fold higher than the control values, respectively. Likewise, restoration of NH_4_^+^ and glutamine to low levels in roots after AVG + Glu treatment induced a significant decrease rather than an increase in nitrate uptake rate.

By contrast, AVG + Glu treatment was associated with a decrease in root/shoot ratio of total AA corresponding to a theoretical supply of 4.8 mM KNO_3_. This glutamate-induced high N status is likely due to the N form used for N uptake and assimilation (e.g. organic *versus* mineral) rather than qualitative changes in individual free amino acid contents *per se*. In this regard, these data confirm a previous work (Leblanc et al., 2013) and indicate that a high glutamate supply inhibits nitrate uptake and increases ^15^NGlu uptake and translocation. Taken together, the results also suggest that Glu induces a general switch in the seedling N status regulating nitrate uptake and AA biosynthesis independently or in parallel to AVG effects.

### Plants Treated With AVG Mimic Some Root Morphological and Biochemical Traits of Mine/Pdx3 Mutants

Because AVG is a strong inhibitor of *TAA1/TAR* activity and roots elongation in dose-dependent manner (Leblanc et al., 2008; Soeno et al., 2010), the lack of tryptophan (Trp) accumulation after AVG treatment is very intriguing (**Figure 1**). This could explain either from ethylene-directed IAA biosynthesis (He et al., 2011; Kim et al., 2018) and/or diversion of Trp flow to other Trp-dependent IAA biosynthetic pathways (Wang et al., 2015). Indeed, transcription factor EIN3, an ethylene-signaling component, is a major inducer of local IAA biosynthesis pathway *via* the transcription of α- and β-subunits of anthranilate synthase (*AnS*) and *TAA1/TARs* genes (He et al., 2011). Therefore, AVG inhibition of ethylene biosynthesis could block the transcription of EIN3, which in turn may impede the Trp biosynthesis pathway and IAA production *via AnS*. Similarly, ethylene-directed IAA biosynthesis is also dependant of pyridoxine 5’-phosphate/pyridoxamine 5’-phosphate (PNP/PMP) oxidase (*PDX3*), an enzyme involved in pyridoxal-5’-phosphate (PLP) salvage pathway and PLP homeostasis (Colinas et al., 2016; Kim et al., 2018). Indeed, in dark-grown condition, root IAA treatment restores the ethylene insensitivity of *mine/pdx3* mutants suggesting that lack of PLP or increase imbalance in the ratio of PMP/PLP contents in the mutants impedes activity of *TAA1/TAR* necessary to produce IAA in roots (Kim et al., 2018).

Intriguingly, *mine/pdx3* mutants that develop under normal conditions show strong inhibition of root growth but not shoot growth (Kim et al., 2018). This result is very similar to data obtained with AVG treatment. In fact, the differential inhibition of AVG can be explained by a diversion of Trp flow to other Trp-dependent IAA biosynthetic pathways in shoots to produce IAA required for growth. Because in the roots, these alternative pathways could be less or not used, this would lead to over accumulation of Trp. This explanation is reinforced by the significant relationships found between Trp contents with the N translocation and accumulation (**Figure 7B**) and morphological variables in shoots (**Figure S1B**). Such relationships have never been found with roots. This also supports the fact that in different species such as corn, wheat, rice and *Arabidopsis* TAA/TARs mutants induced pleiotropic auxin-related processes associated with shoots and roots growth (Philipps et al., 2011; Ma et al., 2014; Yoshikawa et al., 2014; Shao et al., 2017). For example, in maize, TAA (*vt2*) mutants are more affected in their reproductive phase than their vegetative phase (Philipps et al., 2011) and in *Arabidopsis*, the root growth of *tar2* mutants is more altered than the shoots growth (Ma et al., 2014). Furthermore, in *mine/pdx3* mutants, AA were over accumulated in rosette leaves (Colinas et al., 2016) as in N deprivation condition (**Figure 4A**; Le Ny et al., 2013) or after a treatment with AVG (this study). Taken together these data suggest that catalytic inhibition of PLP-dependent aminotransferases by AVG strongly mimics the AA metabolism impairment observed in *mine/pdx3* mutants.

### How to Explain the High Accumulation of His Contents After AVG Treatment

**Figure 10:**
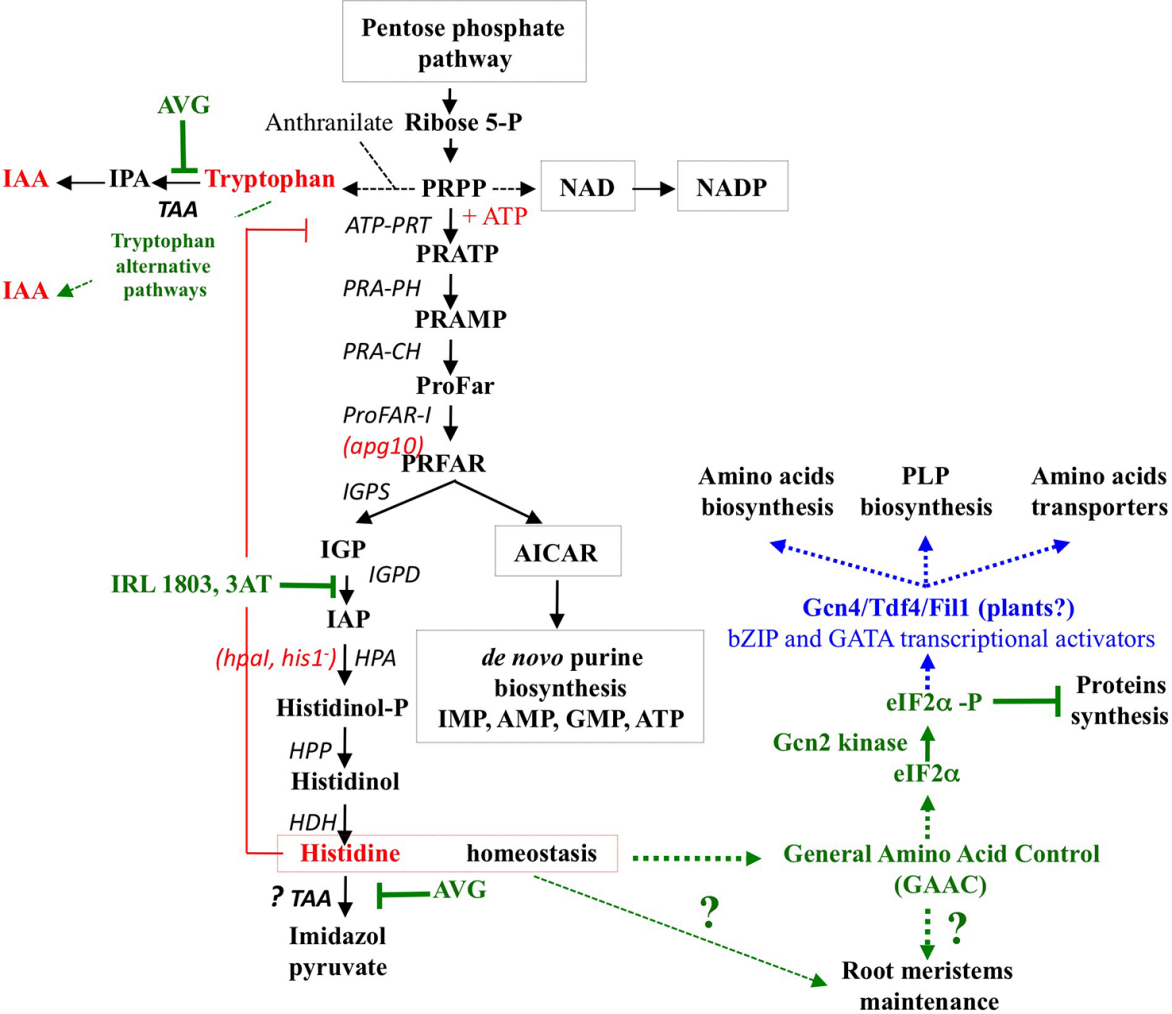
Inhibitors and mutants of the histidine biosynthesis pathway. Enzyme names are shown in black letters with the mutant name in red letters in parentheses. Inhibitors of Histidine biosynthesis and catabolism are designated in green letters and their effects on other metabolic or signalling pathways are indicated by green dotted arrows. 3AT: 3-aminotriazole; *ATP-PRT*: ATP-phosphoribosyl-transferase, *PRA-PH:* Phosphoribosyl-ATP Pyrophosphohydrolase, *PRA-PH* Phosphoribosyl-AMP Cyclohydrolase, *ProFAR-I:* N´-[(5´-phosphoribosyl)formimino]-5-aminoimidazole-4-carboxamide ribonucleotide isomerase, *IGPS*: Imidazole glycerol-phosphate synthase, *IGPD*: Imidazole glycerol-phosphate dehydratase, *HPA*: Histidinol-phosphate aminotransferase. *HPP*: Histidinol-phosphate phosphatase, *HDH*: Histidinol dehydrogenase, *TAA*: Tryptophan aminotransferase. Missing actors in Gcn2 kinase signaling in plants are indicated in blue. (adapted from Ingle, 2011 and Immanuel et al., 2012).

In both sets of experiments (**Figures 8A, B** and **S5**), His contents showed the highest increase compared to other AA in the shoots and roots (by 2.5- and 8-fold, respectively). Because histidinol-phosphate aminotransferase (*HPA*), the last enzyme of His biosynthesis, is a potential target of AVG (**Figure S2**), the increased levels of His were unexpected. Two non-mutually exclusive biochemical hypotheses could explain this result: (i) a diversion of 5’-phosphoribosyl-1-pyrophosphate (PRPP) for His biosynthesis and (ii) an inhibition of His catabolism pathway *via* the involvement of *TAA1/TAR* aminotransferases (**Figure 10**). The first hypothesis could be explained by a cross-regulation mechanism in the metabolic pathway of His and Trp (Stepansky and Leustek, 2006). Indeed, His is metabolically the only other amino acid with Trp (**Figure S2**) that derives a part of its skeleton from PRPP. PRPP is required in tryptophan biosynthetic pathway for the conversion of anthranilate to N-(5’-phosphoribosyl)-anthranilate *via* phosphoribosyl-anthranilate transferase (Pendyala and Wellman, 1975; Last et al., 1991). Therefore, strong increase in His levels of the roots and shoots in response to 10 μM AVG would be caused by a diversion of PRPP required for His biosynthesis, nucleotides production and biosynthesis of NAD and NADP cofactors instead of Trp biosynthesis (**Figure S1** and **Figure 10**). This cross-pathway between His and Trp metabolism was confirmed in *Arabidopsis* by inhibition of imidazole glycerol-phosphate dehydratase (*IGPD*) catalyzing the sixth step of His biosynthesis (Guyer et al., 1995) and in *Nicotiana* by the over-expression of PRPP synthase (Koslowsky et al., 2008).

The second hypothesis could be provided by the promiscuity in TAA activity. In other words, the ability of TAA to catalyse a fortuitous side reaction such as His catabolism in addition to its main reaction. This hypothesis is also supported by the fact that the pathway of His catabolism has not yet been elucidated in plants (Ingle, 2011; Hildebrandt et al., 2015). Thus, in *C. glabrata* and *S. cerevisiae*, Brunke et al. (2014) have recently demonstrated that *TAA1/TARs* aminotransferases also catalyse the conversion of L-histidine and α–ketoglutarate into imidazol-5yl-pyruvate and glutamate (**Figures 10**, **S2**). Since the enzyme activity of cytosolic *TAA1/TARs* is strongly inhibited by AVG (Soeno et al., 2010), this second hypothesis would explain the high accumulation of His. This explanation is also corroborated by high levels of His accumulation in roots and shoots, whereas Trp-dependent pathways other than IPA pathway are only used in shoots to maintain growth. Furthermore, this hypothesis would reinforce the cross-pathway regulation of enzymes in the biosynthesis of Trp and His already observed in plants and fungi (Carsiotis and Jones, 1974; Carsiotis et al., 1974; Niederberger et al., 1981; Hinnebusch, 2005; Stepansky and Leustek, 2006).

### Are AVG and Rhizobitoxine Secreted by Symbiotic and Pathogen Bacteria Only Involved in the Inhibition of Aminocyclopropane-1-Carboxylate Synthase?

Ethylene biosynthesis inhibition by AVG and Rhizobitoxine plays a major role in establishing symbiosis and increasing the nodule number between rhizobacteria and their host legumes (Berkowitz et al., 2006; Boibessot et al., 2016). However, N deprivation or low nitrate concentrations (<50 mg/L = 0.5 mM) are commonly used with AVG treatments to induce nodulation (Xia et al., 2017). Indeed, reduction of nitrogen supply such as nitrate significantly increases the number and weight of nodules (Gibson and Harper, 1985; Macduff et al., 1996; Gan et al, 2004). The nitrogen limitation of AVG-treated seedlings from 1 mM nitrate to 0.2 mM (expressed as N status) indicates that the increase in nodule numbers induced by 10 μM AVG could be caused by both deprivation of N and inhibition of ethylene biosynthesis (Peters and Crist-Estes, 1989). This suggests that the major effects of AVG and Rhizobitoxine secreted by rhizobacteria and pathogenic bacteria on the N metabolism of their host legumes have been overlooked. In this respect, *Lotus* NIN (Nodule INception) transcription factors have been shown to be involved in early-steps of N-regulated symbiosis between *rhizobia* and legumes roots (Schauser et al., 1999; Liu et al., 2019). In *Arabidopsis*, NIN homologous protein: NLP7 (Nin Like Protein 7) is involved in the control of nitrate and N starvation response (Castaings et al., 2009).

### Is AVG-Induced Remodeling of Plant Nitrogen Status due to Inhibition of a Major N-Sensory System?

Induction of AA imbalance associated with nitrate starvation response suggest that the suicide inhibition by AVG of PLP-dependent enzymes probably mimics or impairs a major plant N sensory system. Since Histidine biosynthesis has the highest energy cost among AA and His contents exhibit the greatest variations in plantlets treated with AVG, it is tempting to assign a regulatory role of His homeostasis in these responses. Indeed, previous studies with mutant of different enzymes of His biosynthesis have demonstrated that His homeostasis is crucial for root meristem maintenance, root development and plant growth (El Malki and Jacobs, 2001; Noutoshi et al., 2005; Mo et al., 2006). Similarly, inhibition by the IRL1803 herbicide of *IGPD* catalysing the sixth step of His biosynthesis (**Figure 10**) increased the transcription of eight genes involved in the synthesis of aromatic AA, His, Lys and purines (Guyer et al., 1995). This suggests a cross-pathway regulation of AA biosynthesis genes *via* a general amino acid control system (GAAC) as in yeast treated by another *IGPD* inhibitor: 3-aminotriazole (3-AT) (Hinnebusch, 1984; Natarajan et al., 2001). Although most of the components of GAAC system mediated by the Gcn2 kinase signaling (General Control Non-derepressible-2) are conserved in all eukaryotes (Zhang et al., 2003; Zhang et al., 2008; Lageix et al., 2008; Casthilo et al., 2014), the bZIP transcription factor Gnc4/Atf4 is always the missing component of this N sensory system in plants (Lam et al., 2006; Hey et al., 2010; Immanuel et al., 2012). Recently, Fil1 a transcription factor belonging to GATA family and non-orthologous to GCN4/ATF4 bZIP family has been discovered in *Schizosaccharomyces pombe* (Duncan et al., 2018). In plants, two other transcription factors of GATA family such as NTL1 (NiT-2-Like) and GNC (GATA, Nitrate-inducible, Carbon metabolism-involved) are already involved in nitrate assimilation and glucose responses (Reyes et al., 2004; Bi et al., 2005).

In conclusion, AVG is not a sufficiently reliable inhibitor to validate the genetic processes controlling the effects of ethylene and ethylene/auxin crosstalk on the root and shoot growth when its concentrations exceed 5 μM (Rahman et al, 2000; Okamoto et al., 2008; Leblanc et al., 2008; Le Deunff and Lecourt, 2016; Le Deunff 2018). As shown in this study, 10 μM AVG treatment induces a N limitation condition close to deprivation associated with an increase of nitrate uptake and translocation, imbalance in AA metabolism and a deregulation of His homeostasis. These effects appear to be associated with differential use of Trp in roots and shoots to produce IAA through other biosynthetic pathways that do not utilize PLP-dependent enzymes. Therefore, the use of high AVG concentrations range could be an original and valuable tool to highlight, by using RNAseq and metabolic profiling, the major N sensory system that is involved in reprogramming aminotranferases network associated with the root and shoot morphological changes.

## Data Availability Statement

All datasets generated for this study are included in the article/**Supplementary Material**.

## Author Contributions

EL and JL designed the research. EL, JL, PB, and CD performed the experiments. CD performed UPLC analyses. EL and CD analyzed the data. EL, JL, and CD wrote the paper.

## Conflict of Interest

The authors declare that the research was conducted in the absence of any commercial or financial relationships that could be construed as a potential conflict of interest.
