# Supplementary material for: Inhibition of Aminotransferases by Aminoethoxyvinylglycine Triggers a Nitrogen Limitation Condition and Deregulation of Histidine Homeostasis That Impact Root and Shoot Development and Nitrate Uptake
Source: Front Plant Sci. 2019 Nov 7;10:1387. doi: 10.3389/fpls.2019.01387 (PMC6855093; doi:10.3389/fpls.2019.01387)
Supplement: Supplementary file 1 [file Presentation_1.pptx]

## Slide 1
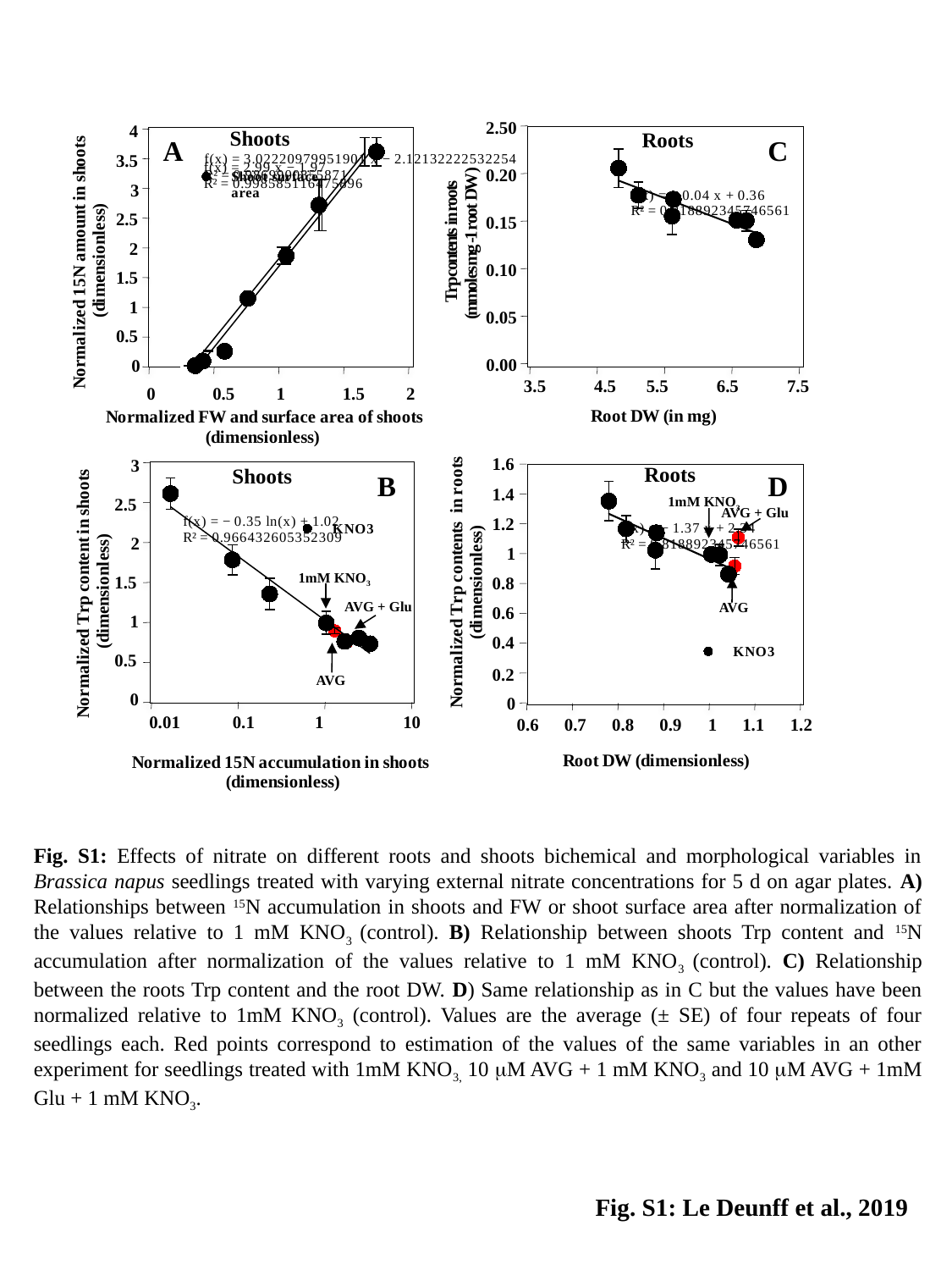

### Chart
| Category | |
|---|---|C
### Chart
| Category | | |
|---|---|---|A
2.50
0.20
0.15
0.10
0.05
0.00
Shoots
4
3.5
3
2.5
2
1.5
1
0.5
0
Roots
3.5
4.5
5.5
6.5
7.5
0
0.5
1
1.5
2
### Chart
| Category | | |
|---|---|---|
### Chart
| Category | | |
|---|---|---|B
1.6
1.4
1.2
1
0.8
0.6
0.4
0.2
0
3
2.5
2
1.5
1
0.5
0
Roots
Shoots
D
1mM KNO3
AVG + Glu
1mM KNO3
AVG + Glu
AVG
AVG
0.01
0.1
1
10
0.6
0.7
0.8
0.9
1
1.1
1.2
Fig. S1: Effects of nitrate on different roots and shoots bichemical and morphological variables in Brassica napus seedlings treated with varying external nitrate concentrations for 5 d on agar plates. A) Relationships between 15N accumulation in shoots and FW or shoot surface area after normalization of the values relative to 1 mM KNO3 (control). B) Relationship between shoots Trp content and 15N accumulation after normalization of the values relative to 1 mM KNO3 (control). C) Relationship between the roots Trp content and the root DW. D) Same relationship as in C but the values have been normalized relative to 1mM KNO3 (control). Values are the average (± SE) of four repeats of four seedlings each. Red points correspond to estimation of the values of the same variables in an other experiment for seedlings treated with 1mM KNO3, 10 mM AVG + 1 mM KNO3 and 10 mM AVG + 1mM Glu + 1 mM KNO3.
Fig. S1: Le Deunff et al., 2019
