# Supplementary material for: Inhibition of Aminotransferases by Aminoethoxyvinylglycine Triggers a Nitrogen Limitation Condition and Deregulation of Histidine Homeostasis That Impact Root and Shoot Development and Nitrate Uptake
Source: Front Plant Sci. 2019 Nov 7;10:1387. doi: 10.3389/fpls.2019.01387 (PMC6855093; doi:10.3389/fpls.2019.01387)
Supplement: Supplementary file 2 [file Presentation_2.pptx]

## Slide 1
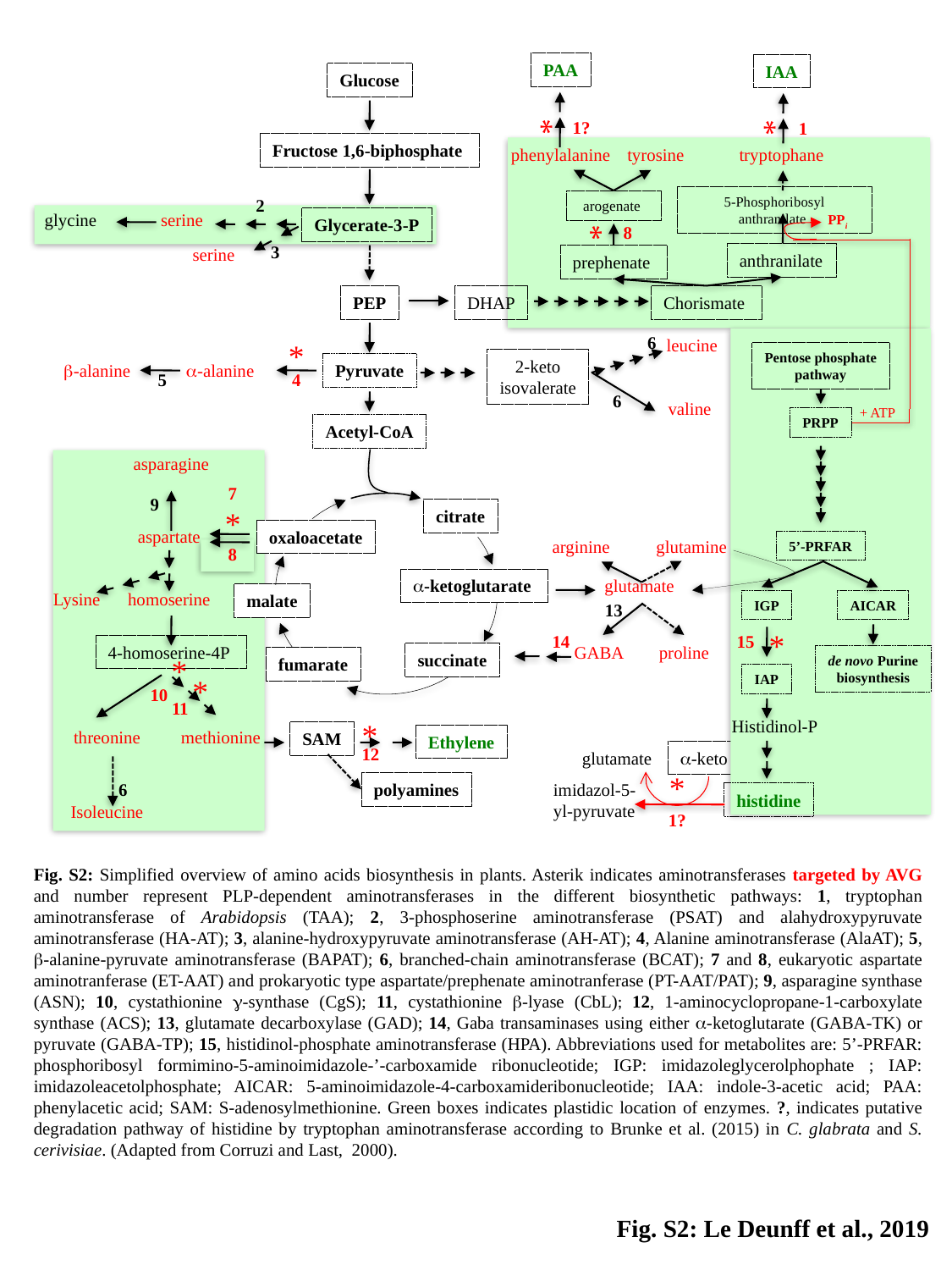

PAA
IAA
Glucose
*
*
1?
1
Fructose 1,6-biphosphate
phenylalanine
tyrosine
tryptophane
5-Phosphoribosyl anthranilate
2
arogenate
glycine
serine
PPi
*
Glycerate-3-P
8
3
serine
anthranilate
prephenate
PEP
DHAP
Chorismate
6
leucine
*
Pentose phosphate
pathway
PRPP
5’-PRFAR
2-keto
isovalerate
b-alanine
a-alanine
Pyruvate
5
4
6
valine
+ ATP
Acetyl-CoA
asparagine
7
9
*
citrate
aspartate
oxaloacetate
arginine
glutamine
8
a-ketoglutarate
glutamate
homoserine
Lysine
malate
IGP
AICAR
13
*
15
14
4-homoserine-4P
GABA
proline
succinate
*
de novo Purine
biosynthesis
fumarate
IAP
*
10
11
*
Histidinol-P
threonine
methionine
SAM
Ethylene
12
glutamate
a-keto
*
1?
6
polyamines
imidazol-5-
yl-pyruvate
histidine
Isoleucine
Fig. S2: Simplified overview of amino acids biosynthesis in plants. Asterik indicates aminotransferases targeted by AVG and number represent PLP-dependent aminotransferases in the different biosynthetic pathways: 1, tryptophan aminotransferase of Arabidopsis (TAA); 2, 3-phosphoserine aminotransferase (PSAT) and alahydroxypyruvate aminotransferase (HA-AT); 3, alanine-hydroxypyruvate aminotransferase (AH-AT); 4, Alanine aminotransferase (AlaAT); 5, b-alanine-pyruvate aminotransferase (BAPAT); 6, branched-chain aminotransferase (BCAT); 7 and 8, eukaryotic aspartate aminotranferase (ET-AAT) and prokaryotic type aspartate/prephenate aminotranferase (PT-AAT/PAT); 9, asparagine synthase (ASN); 10, cystathionine g-synthase (CgS); 11, cystathionine b-lyase (CbL); 12, 1-aminocyclopropane-1-carboxylate synthase (ACS); 13, glutamate decarboxylase (GAD); 14, Gaba transaminases using either a-ketoglutarate (GABA-TK) or pyruvate (GABA-TP); 15, histidinol-phosphate aminotransferase (HPA). Abbreviations used for metabolites are: 5’-PRFAR: phosphoribosyl formimino-5-aminoimidazole-’-carboxamide ribonucleotide; IGP: imidazoleglycerolphophate ; IAP: imidazoleacetolphosphate; AICAR: 5-aminoimidazole-4-carboxamideribonucleotide; IAA: indole-3-acetic acid; PAA: phenylacetic acid; SAM: S-adenosylmethionine. Green boxes indicates plastidic location of enzymes. ?, indicates putative degradation pathway of histidine by tryptophan aminotransferase according to Brunke et al. (2015) in C. glabrata and S. cerivisiae. (Adapted from Corruzi and Last, 2000).
Fig. S2: Le Deunff et al., 2019
