# Supplementary material for: Inhibition of Aminotransferases by Aminoethoxyvinylglycine Triggers a Nitrogen Limitation Condition and Deregulation of Histidine Homeostasis That Impact Root and Shoot Development and Nitrate Uptake
Source: Front Plant Sci. 2019 Nov 7;10:1387. doi: 10.3389/fpls.2019.01387 (PMC6855093; doi:10.3389/fpls.2019.01387)
Supplement: Supplementary file 4 [file Presentation_4.pptx]

## Slide 1
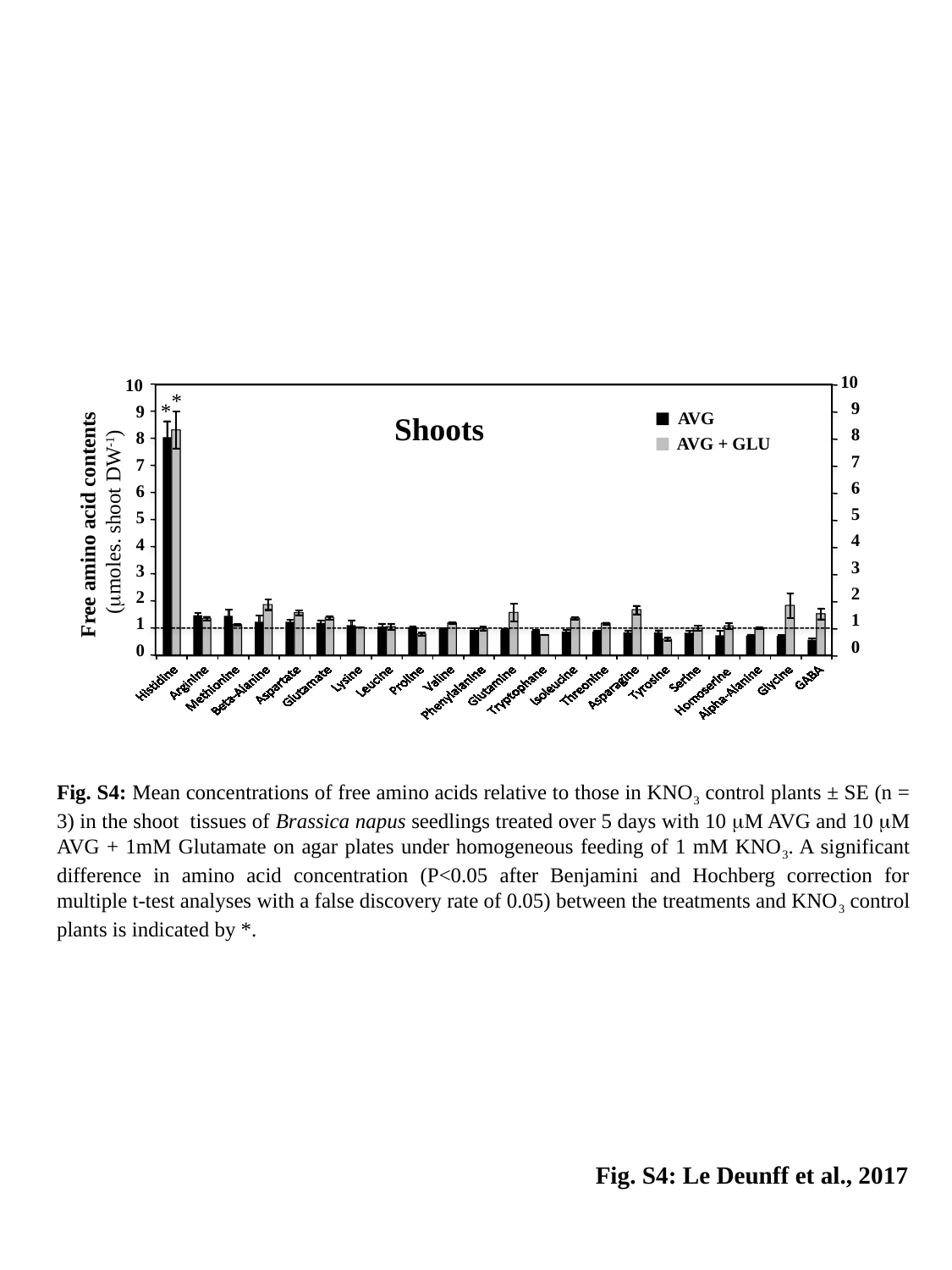

10
9
8
7
6
5
4
3
2
1
0
10
9
8
7
6
5
4
3
2
1
0
*
*
Shoots
AVG
AVG + GLU
Free amino acid contents
(mmoles. shoot DW-1)
Fig. S4: Mean concentrations of free amino acids relative to those in KNO3 control plants ± SE (n = 3) in the shoot tissues of Brassica napus seedlings treated over 5 days with 10 mM AVG and 10 mM AVG + 1mM Glutamate on agar plates under homogeneous feeding of 1 mM KNO3. A significant difference in amino acid concentration (P<0.05 after Benjamini and Hochberg correction for multiple t-test analyses with a false discovery rate of 0.05) between the treatments and KNO3 control plants is indicated by *.
Fig. S4: Le Deunff et al., 2017
