# Supplementary material for: Inhibition of Aminotransferases by Aminoethoxyvinylglycine Triggers a Nitrogen Limitation Condition and Deregulation of Histidine Homeostasis That Impact Root and Shoot Development and Nitrate Uptake
Source: Front Plant Sci. 2019 Nov 7;10:1387. doi: 10.3389/fpls.2019.01387 (PMC6855093; doi:10.3389/fpls.2019.01387)
Supplement: Supplementary file 5 [file Presentation_5.pptx]

## Slide 1
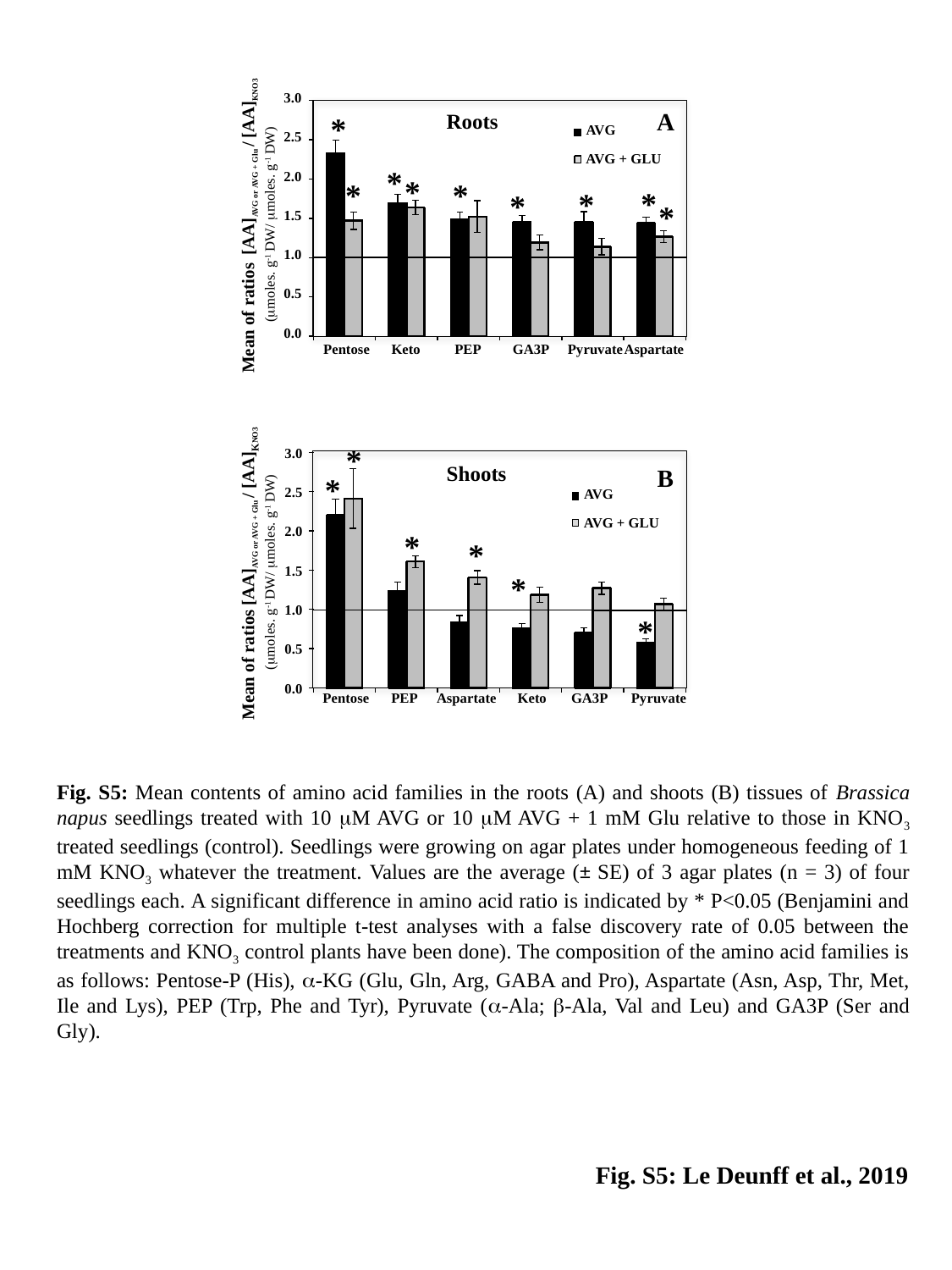

3.0
2.5
2.0
1.5
1.0
0.5
0.0
A
Roots
AVG
AVG + GLU
Mean of ratios [AA]AVG or AVG + Glu / [AA]KNO3
(mmoles. g-1 DW/ mmoles. g-1 DW)
Pentose
Keto
PEP
GA3P
Pyruvate
Aspartate
*
*
*
*
*
*
*
*
*
3.0
2.5
2.0
1.5
1.0
0.5
0.0
Shoots
B
AVG
AVG + GLU
Mean of ratios [AA]AVG or AVG + Glu / [AA]KNO3
(mmoles. g-1 DW/ mmoles. g-1 DW)
Pentose
PEP
Aspartate
Keto
GA3P
Pyruvate
*
*
*
*
*
*
Fig. S5: Mean contents of amino acid families in the roots (A) and shoots (B) tissues of Brassica napus seedlings treated with 10 mM AVG or 10 mM AVG + 1 mM Glu relative to those in KNO3 treated seedlings (control). Seedlings were growing on agar plates under homogeneous feeding of 1 mM KNO3 whatever the treatment. Values are the average (± SE) of 3 agar plates (n = 3) of four seedlings each. A significant difference in amino acid ratio is indicated by * P<0.05 (Benjamini and Hochberg correction for multiple t-test analyses with a false discovery rate of 0.05 between the treatments and KNO3 control plants have been done). The composition of the amino acid families is as follows: Pentose-P (His), a-KG (Glu, Gln, Arg, GABA and Pro), Aspartate (Asn, Asp, Thr, Met, Ile and Lys), PEP (Trp, Phe and Tyr), Pyruvate (a-Ala; b-Ala, Val and Leu) and GA3P (Ser and Gly).
Fig. S5: Le Deunff et al., 2019
